# Bayesian Detection of Causal Rare Variants under Posterior Consistency

**DOI:** 10.1371/journal.pone.0069633

**Published:** 2013-07-26

**Authors:** Faming Liang, Momiao Xiong

**Affiliations:** 1 Department of Statistics, Texas A&M University, College Station, Texas, United States of America; 2 Division of Biostatistics, University of Texas School of Public Health, Houston, Texas, United States of America; University of Southern California, United States of America

## Abstract

Identification of causal rare variants that are associated with complex traits poses a central challenge on genome-wide association studies. However, most current research focuses only on testing the global association whether the rare variants in a given genomic region are collectively associated with the trait. Although some recent work, e.g., the Bayesian risk index method, have tried to address this problem, it is unclear whether the causal rare variants can be consistently identified by them in the small-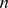-large-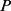 situation. We develop a new Bayesian method, the so-called Bayesian Rare Variant Detector (BRVD), to tackle this problem. The new method simultaneously addresses two issues: (i) (Global association test) Are there any of the variants associated with the disease, and (ii) (Causal variant detection) Which variants, if any, are driving the association. The BRVD ensures the causal rare variants to be consistently identified in the small-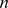-large-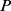 situation by imposing some appropriate prior distributions on the model and model specific parameters. The numerical results indicate that the BRVD is more powerful for testing the global association than the existing methods, such as the combined multivariate and collapsing test, weighted sum statistic test, RARECOVER, sequence kernel association test, and Bayesian risk index, and also more powerful for identification of causal rare variants than the Bayesian risk index method. The BRVD has also been successfully applied to the Early-Onset Myocardial Infarction (EOMI) Exome Sequence Data. It identified a few causal rare variants that have been verified in the literature.

## Introduction

Testing the phenotypic association of millions of individual SNPs across the genome has been one of the major goals of the genome-wide association study (GWAS). To date, hundreds of putative disease gene loci have been detected based on the common disease common variant assumption. However, the detected genetic variants typically account for only a small fraction of disease heritability. Nowadays, it has been widely acknowledged that the missing disease heritability may be due to rare variants. Many studies show that the rare variants tend to have larger effects than common variants. As pointed out in [1], most rare variants can have much greater odds ratio than common variants, and many non-synonymous rare mutations from exon sequencing are functional variants for some common diseases. The rare variant effects have been investigated in some studies. For example, [2] found that the rare variants in the IFIH1 gene are strongly associated with Type I diabetes, and [3] found that multiple rare variants in NPC1L1 are associated with reduced sterol absorption and plasma low density lipoprotein levels. Therefore, development of statistical methods that are powerful enough to detect causal rare variants has become essential for the GWAS.

The statistical power of genetic variant detection depends on the sample size, the variant effect and the minor allele frequency (MAF). Since the MAF of the rare variant is low, the single variant testing-based methods, such as the 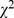-test and Fisher's exact test, that are traditionally used in common variant association studies, tend to have a low power. To address this issue, methods that test the collective effect of rare variants for a given genomic region have been developed, see e.g., the combined multivariate and collapsing (CMC) test [4], weighted sum statistic (WSS) test [5], and sequence kernel association test (SKAT) [6]. The CMC and WSS tests are variant pooling methods, in which the rare variants are collapsed or summed into a super-variant and then the disease association is tested with this super-variant. Their power can depend on the weighting scheme they employed, which often emphasizes low frequency alleles in controls. Numerous alternative methods [7], [8] are largely their variations. The SKAT test is developed based on random effect models, which assumes a common distribution for the genetic effects of variants at different sites and tests for the null hypothesis that the distribution has zero variation.

Although testing the collective effects of rare variants is challenging, identifications of the rare variants which, if any, are driving the association (i.e., the so-called causal rare variants) is even more challenging and scientifically more interesting. Along this research direction, some methods have been developed, e.g., the RARECOVER method [9], variable threshold (VT) method [10], evolutionary mixed model for pooled association testing (EMMPAT) method [11], hierarchical generalized linear model (HGLM) method [12], [13], and Bayesian risk index (BRI) method [14]. The RARECOVER method uses a greedy search algorithm to determine an association set of variants. The VT method selects all variants with the MAF lower than a varying threshold to be included in the association set. The RARECOVER and VT focus mainly on the global association test and lack a formal test to determine the marginal effect of each variant, and thus are unable to formally determine which variants are most likely driving the association. The EMMPAT simultaneously evaluates the effects of all variants under the framework of mixed effect models. This is similar to HGLM, where the regression coefficients are simultaneously estimated for all variants. As a consequence of the simultaneous parameter estimation, when the number of variants is greater than the number of subjects, the variant effects evaluated by EMMPAT and HGLM might not be very reliable due to the multicollinearity of variants. The BRI is a Bayesian method, which can evaluate the marginal effect of each variant by allowing for uncertainty into which variants are included in the association set.

While BRI has made a solid step toward detection of causal rare variants, it is unclear whether it can identify causal rare variants consistently for small-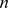-large-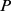 problems, in which the number of variants can be much greater than the number of subjects. In addition, BRI assumes the effect of each causal variant to be the same. Since this is not true for real problems, the performance of BRI may be sub-optimal. In this paper, we propose a new Bayesian method, the so-called Bayesian Rare Variant Detector (BRVD), for identification of causal rare variants. The new method simultaneously answers two questions:

- (Global association test) Are there any of the variants associated with the disease?
- (Causal variant detection) Which variants, if any, are driving the association?

The BRVD ensures the causal rare variants to be consistently identified in the small-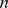-large-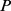 situation by imposing some appropriate prior distributions on the model and model specific parameters. In addition, to enhance detection of causal rare variants, the BRVD specifies for each variant a different prior selection probability (or weight) which is adversely proportional to its MAF. To accelerate the computation, we also propose a parallel version of BRVD based on the strategy of divide-and-conquer. The parallel BRVD has an embarrassingly parallel structure and can be conveniently applied to the problems for which the number of variants is extremely large. Our numerical results indicate that the BRVD can be more powerful for testing the global association than the existing methods, such as CMC, WSS, SKAT, C-alpha, RARECOVER, VT, and BRI, and more powerful than BRI for identification of causal rare variants. The BRVD has also been successfully applied to the early-onset myocardial infarction (EOMI) data: It identified a few causal rare variants that have been verified in the literature.

## Materials and Methods

### The global association test and Bayesian factor

Assume that 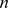 subjects are sequenced in a genomic region with 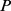 SNPs. Let 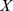 be a 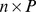 genotype matrix coded as 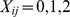 for the number of copies of the minor allele measured for individual 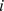 at SNP 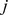, let 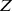 be a 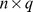 matrix of covariates, e.g., age and race, and let 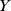 be a 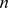-dimensional binary vector indicating the disease status of the 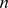 subjects. The BRVD uses a logistic regression model to relate the covariates and a subset of variants to the disease status variable. Let 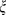 denote a subset of variants, and let 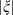 denote the number of variants included in 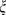. Let 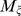 denote the logistic regression model corresponding to the subset 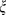, which can be expressed as
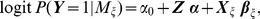
(1)where 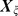 denotes the genotype matrix corresponding to the subset 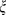, and 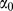, 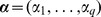 and 
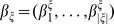
 are the regression coefficients. For this model, the global association test is to test the hypotheses
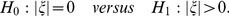
(2)Let 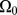 denote the parameter space of the null model 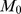, i.e., the domain of the parameters 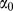 and 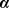. Let 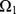 denote the parameter space of the alternative models, which can be expressed as 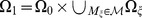, where 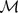 denotes the set of all possible models with 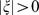 and 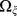 is the domain of 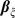.

Let 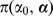 denote the prior distribution of 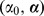, let 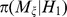 denote the prior probability imposed on the model 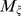 under the hypothesis 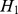, and let 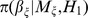 denote the prior distribution of 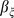. Then the Bayesian factor for the test (2) can be expressed as
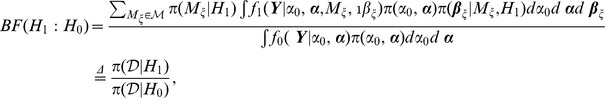
(3)where 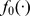 and 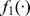 denote the likelihood functions of the null and alternative models, respectively; 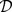 denotes the data; and 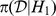 and 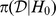 are the Bayesian evidence corresponding to the hypotheses 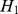 and 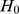, respectively. As in [14], [15], (3) can also be expressed as the weighted average of the individual Bayes factors for comparing each model in 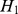 to the null model 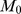 with the weights given by the prior probability 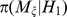; that is,
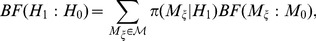
(4)where 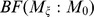 is defined as the ratio of 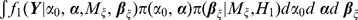 and 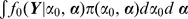. Let 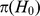 denote the prior probability imposed on the null model, and let 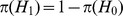 denote the total prior probabilities imposed on the alternative models. Then the respective posterior probabilities of 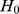 and 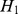 are given by
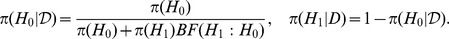
A value of BF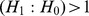 means that the alternative hypothesis is more strongly supported by the data under consideration than the null hypothesis. Harold Jeffreys [16] gave a scale, which is reproduced in Table 1, for interpretation of Bayes factors. Decisions about which hypothesis is more likely true can be made based on the scale of Bayes factors.

**Table 1:** Jeffrey's grades of evidence (Jeffreys, 1961).

Grade	BF(*H*_1_:*H*_0_)	*π*(*H*_1_|*D*)	Evidence against *H*_0_
1	1∼3	0.50∼0.75	Barely worth mentioning
2	3∼10	0.75∼0.91	Substantial
3	10∼30	0.91∼0.97	Strong
4	30∼100	0.97∼0.99	Very strong
5	>100	>0.99	Decisive

The posterior probability 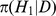 is calculated with the prior probabilities 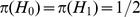.

The Bayes factor (3) depends on the prior distributions, 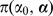, 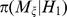, and 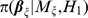. In particular, the dependence on the model prior 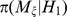 can be substantial. This inevitably leads to ambiguity in interpretation of Bayes factors. To minimize the ambiguity, we suggest to choose the priors 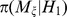 and 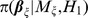 such that the Bayesian evidence of 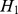 is maximized. The resulting prior is the so-called type-II maximum likelihood prior [17]. Since maximizing the evidence over general priors is impossible, we further suggest to maximize the evidence over a specified class of priors. This will be detailed below. We note that a similar strategy has been suggested in [18] for testing a point null hypothesis. Since 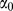 and 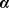 are common parameters for all models, 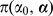 is fixed to a Gaussian-truncated-inverse-gamma prior in all simulations of this paper.

### The prior and posterior distributions

Let 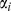, 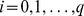, be subject to the independent Gaussian prior:
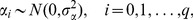
(5)where the variance 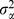 is subject to a truncated inverse-gamma prior
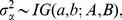
(6)defined on the interval 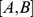, where 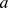 and 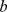 are the shape and scale parameters, respectively. The density function of (6) is given by
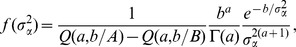
where 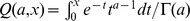 is an incomplete gamma function and can be evaluated numerically. In the literature, 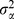 is usually assumed an inverse-gamma prior distribution. Here 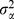 is restricted to take values from the bounded interval 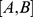. As shown in Lemma 1 of File S1 (Section S1), this restriction plays an important role in establishing the posterior consistency [19], [20] for the model (1). The posterior consistency means the true density of 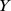 can be estimated consistently by the density of 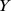 under the models sampled from the posterior distribution. For the same reason, we let 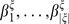 be subject to the independent Gaussian prior
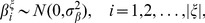
(7)with the variance 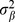 being subject to the truncated inverse-gamma prior 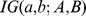. For simplicity of computation, we further assume 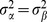; that is, 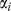 and 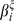 have the same prior variance.

Let 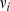 denote the prior selection probability of variant 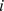. Let 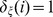 if variant 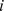 is included in the subset 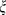 and 0 otherwise. The prior probability of the model 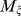 under 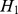 is given by
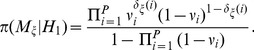
(8)To enhance selection of causal rare variants, we suggest to set 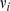 as a decreasing function of MAF. In this paper, we set
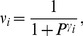
(9)where 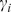 is restricted to the interval 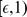 for some constant 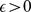. In this paper, we set 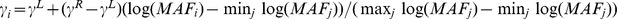, where 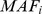 denotes the minor allele frequency of variant 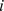, and 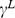 and 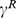 are hyperparameters to be specified by the user. In addition, we fix 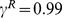 and choose 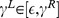 such that the Bayes factor BF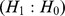 is maximized. Note that (9) is not necessarily optimal. In practice, one may try different settings for 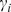 and 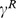.

As shown in File S1 (Section S1), the above prior setting, together with the identifiability condition of the true model, leads to the consistency of causal variant selection. Our priors are different from the conventional “Gaussian–inverse-gamma–beta” priors in two aspects. First, we let 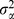 and 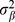 be subject to the truncated inverse-gamma prior, which ensures the eigenvalues of the prior covariance matrix of 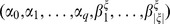 to be bounded. While the boundedness condition cannot be achieved with the inverse-gamma prior. Second, we define 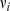 in (9) as a decreasing function of 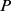. As explained in [21], this is important for variant selection in the small-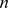-large-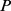 scenario, because it controls for the multiplicity: If 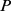 grows large, then 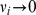. Under appropriate conditions, it can be shown that the resulting *a priori* model size 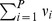 is bounded by a function (of 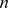) of order 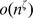 for some 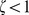. While this condition cannot be satisfied if 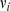 is subject to a beta prior for which both the shape and scale parameters are constants independent of 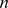.

Let 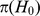 and 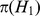 denote the prior probabilities imposed on 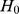 and 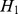, respectively. Then the posterior distribution of the model (1) is given by
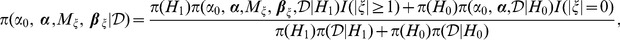
(10)where 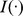 is the indicator function, and 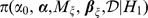 and 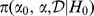 are given in File S1 (Section S0).

In all simulations of this paper, we fixed the hyperparameters 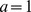, 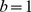, 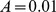, 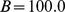, and 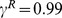. The choice of 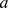, 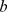, 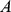 and 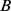 allows 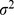 to vary over the interval 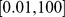 which is large enough for most rare variant selection problems. The only remaining hyperparameter is 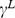, which can be determined by maximizing the Bayes factor BF(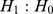) over the interval 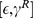. For most examples of this paper, we tried *γ^L^* = 0.4, 0.5, …, 0.9, 0.95, 0.99 or a subset of them.

### Bayes factor estimation

For the global association test, the key step is Bayes factor estimation. As implied by (4), an exact evaluation of the global Bayes factor needs to sum over all models under 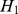. When 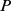 is large, this is prohibitive. For this reason, [14], [15] suggested to replace the sum over the entire model space 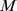 with the sum over the models sampled by a Markov chain Monte Carlo (MCMC) algorithm. However, the resulting estimator is shown to provide only a lower bound for the global Bayes factor. In this paper, we propose to estimate the global Bayes factor using the stochastic approximation Monte Carlo (SAMC) algorithm [22]. The resulting estimator is consistent.

To facilitate the description of the SAMC algorithm, we define the following notations. Let 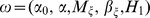 for a model simulated from the posterior distribution (10) under 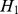, and let 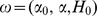 for a model simulated under 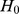. Define
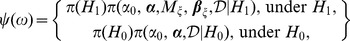
which is the unnormalized posterior distribution of the model (1). Let 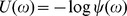, which is called the energy function in terms of physics. To apply the SAMC algorithm to estimate the Bayes factor, we partition the sample space as follows: Treat 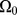 as a single subregion, i.e., setting 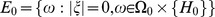, and partition 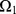 according to the energy function into 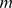 subregions: 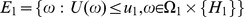, 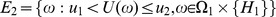, …, 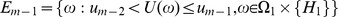, 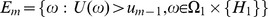, where 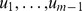 are pre-specified numbers. The sample space 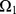 can also be partitioned according to the value of 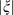. However, when 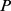 is large, this alternative partition often leads to a slower convergence of SAMC, as which encourages SAMC to sample the models of different sizes instead of those of low energy values.

SAMC seeks to draw samples from each of the subregions with a pre-specified frequency. For the time being, we assume that all the 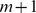 subregions are non-empty; that is, 
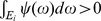
 for 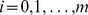. Let 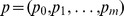 denote the vector of desired sampling frequencies of the 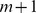 subregions, where 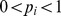 and 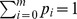. Henceforth, 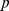 is called the desired sampling distribution. Let 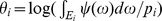 for 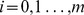, let 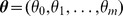, and let 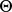 denote the domain of 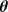. Let 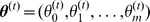 denote the working estimate of 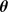 obtained at iteration 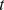. Let 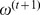 denote a sample drawn at iteration 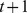 from the MH kernel 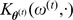, which is constructed with the proposal distribution 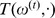 and admits (11) as the invariant distribution:
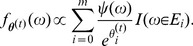
(11)Define 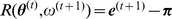, where 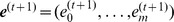 and 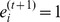 if 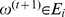 and 0 otherwise. Note that the dependence of 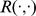 on 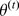 is implicit through the sample 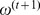. To have the algorithm complied with the notation of stochastic approximation, 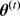 is still included in the function 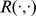. Let 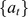 be a positive, non-decreasing sequence satisfying the conditions,
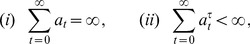
(12)for some 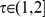. In the context of stochastic approximation, 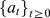 is called the gain factor sequence.

In this paper, we assume that 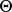 is compact; that is, assuming that the sequence 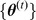 can be kept in a compact set. Extension of this algorithm to the case that 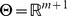 is trivial with the technique of varying truncations studied in [23], [24], which ensures, almost surely, that the sequence 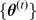 remains in a compact set. In simulations, we can set 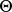 to a huge set, e.g., 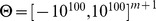, which, as a practical matter, is equivalent to setting 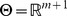. Let 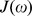 denote the index of the subregion that the sample 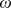 belongs to, which takes values in 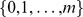. With the above notations, one iteration of SAMC can be described as follows.

### Algorithm 0.1 (The SAMC algorithm)

*(a) (Sampling) Simulate a sample* 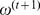 *by a single MH update with the target distribution as defined in (11):*

*(a. 1) Generate* 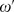 *according to a proposal distribution* 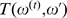 *. Refer to File S1 (Section S2) for the definition of* 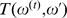 *.*

*(a. 2) Calculate the ratio*

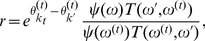
(13)
*where* 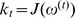 *and* 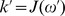 *are the indices of the subregions that* 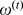 *and* 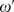 *belong to, respectively.*

*(a. 3) Accept the proposal with probability* 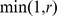 *. If it is accepted, set* 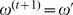 *; otherwise, set* 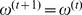 *.*

*(b) (* 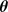 *-updating) Set*

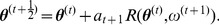
(14)
*If* 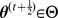 *, set* 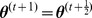 *; otherwise, find a value of* 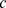 *such that* 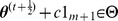 *and set* 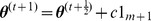 *, where* 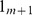 *denotes a constant* 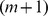 *-vector of ones.*

SAMC is an adaptive MCMC algorithm for which the invariant distribution of the MH kernel changes from iteration to iteration. Due to the adaptive change of the invariant distributions, SAMC possesses a self-adjusting mechanism: If a proposal is rejected, then the sample 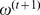 will be retained in the current subregion, the 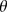-value associated with the current subregion will be adjusted to a larger value, and the overall rejection probability of the next iteration will be reduced. This mechanism warrants the algorithm not to be trapped by local energy minima. The SAMC algorithm represents a significant advance in simulations of complex systems for which the energy landscape is rugged.

The proposal distribution 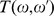 is usually assumed to satisfy the local positive condition: For every 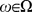, there exist 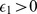 and 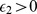 such that
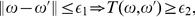
(15)where 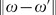 denotes a distance norm between 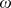 and 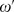. This is a natural condition in MCMC theory. In practice, this kind of proposals can be easily designed for both discrete and continuum systems as discussed in the literature [22]. Regarding the convergence of SAMC, [22] established the following result: Under the conditions (12) and (15) and some regularity conditions, for all non-empty subregions,
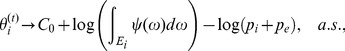
(16)as 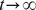, where 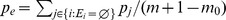, 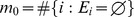 is the number of empty subregions, and 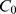 is a constant which can be determined by imposing a constraint on 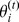, e.g., 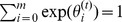.

For global association tests, we set the desired sampling distribution to be uniform, i.e., setting 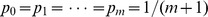. For mathematical simplicity, we have constrained 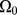 and 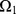 to two large compact sets by restricting 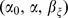 to the set 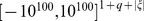, which, as a practical matter, is equivalent to 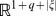. The gain factor sequence 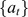 is set in the form
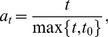
(17)where 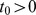 is a user-specified number. It is easy to verify that (17) satisfies the condition (12). A large value of 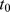 will allow the SAMC sampler to reach all subregions quickly, even when 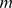 is large. The proposal distribution 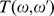 is described in File S1 (Section S2). It is easy to see that it satisfies the condition (15). Then, by (16), we have the following result:
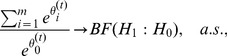
(18)as 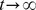. That is, SAMC provides a consistent estimator for the Bayes factor.

### Rare variant detection

In this section, we describe how to detect rare variants when the global association test shows positive support for the hypothesis 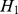.

Identification of important variables based on the marginal inclusion probability has been widely used in Bayesian variable selection, see, for example, [25] for the case of large-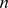-small-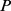 normal linear models, and [26] for small-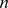-large-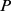 generalized linear models. Let 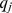 denote the marginal inclusion probability of variable 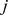. A conventional rule is to choose the variables for which the marginal inclusion probability is greater than a threshold value 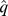; i.e., setting 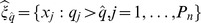 as an estimator of 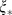, the set of true model variables. Based on [26], we show in Lemma 2 of File S1 (Section S1) that this rule possesses the properties of sure screening and consistency for rare variant detection under the priors given in Section 0. The sure screening property implies that for some choice of 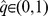,
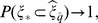
as the sample size 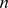 tends to infinity. The property of variant selection consistency implies that
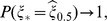
as the sample size 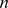 tends to infinity.

To implement the rule 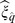 for causal variant detection, one needs a consistent estimator for the marginal inclusion probability under 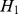 and a method for determining the threshold value 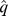. In SAMC, the marginal inclusion probability can be consistently estimated as follows. Let 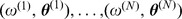 denote the samples drawn by SAMC in a run. Liang [27] showed that SAMC is actually a dynamic importance sampling algorithm and for any integrable function 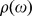, as 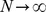,
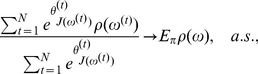
(19)where 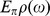 denotes the expectation of 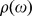 with respect to the target distribution 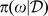. This result implies
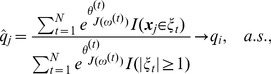
(20)as 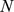 goes to infinity; that is, the estimator 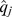 is consistent.

To determine the threshold 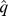, [26] proposed a multiple hypothesis testing-based procedure based on the work [28]. This procedure is adopted in the paper and briefly described in File S1 (Section S3).

### Empirical Power Simulations

To explore the power of the proposed method versus other alternative methods for the global association tests and rare variant detection, we simulated 200 datasets, with 100 simulated under 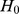 and 100 under 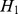. Each dataset consists of 250 cases and 250 controls, and each subject consists of 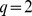 covariates. The first covariate is binary, which mimics the gender of the subjects. The second covariate is drawn uniformly from the interval 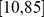, which mimics the age of the subjects. The regression coefficients of the two covariates are set to 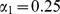 and 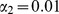, respectively. The genotypes of each subject are simulated by resampling from a haplotype dataset given in the package *SKAT*. The haplotype dataset is generated by the calibrated coalescent model with a mimicking linkage disequilibrium (LD) structure of European ancestry. To emphasize rare events, the variants with MAF greater than 5% have been removed from the haplotype dataset before resampling. For the 100 datasets simulated under 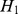, the first 10 variants are assumed to be causal with the regression coefficients given by 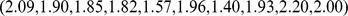, which represents a random sample drawn from 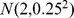. Then we remove the zero-MAF variants from the resampled dataset and keep only the first 600 non-zero MAF variants for further analysis. Because of this deletion step, the number of causal variants becomes a random variable for each dataset. For the 100 datasets simulated under 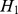, the number of causal variants ranges from 5 to 9, and has a mean value of 7.81 with standard deviation 0.92. The average MAF of the first 9 variants is 0.833% with standard deviation 0.0012. Among the first 9 variants, the maximum MAF is 1.155%. Variants 1 and 2 have very low MAFs, which are 0.183% and 0.293%, respectively. Due to their low MAFs, identification of the causal variants, especially for variants 1 and 2, has put a great challenge on the existing methods.

### Comparison with Other Methods

We compare the BRVD with the competing Bayesian method *Bayesian risk index (BRI)* for both global association tests and causal variant detection. We also compare BRVD with the commonly used non-Bayesian methods, including CMC, WSS, SKAT, and RARECOVER, for global association tests. Among the four non-Bayesian methods, CMC and WSS belong to the class of variant pooling methods, SKAT belongs to the class of random effect model-based methods, and RARECOVER belongs to the class of variable selection methods. These methods can be briefly described as follows.

- Bayesian risk index (BRI) [14]: For a model 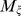, the BRI defines the risk index as the sum of the selected variants, i.e.,

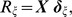
where 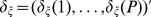 is a binary vector which indicates the variants included in the model 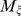. Then it conducts an approximate Bayesian analysis for the model
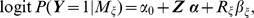
under a Beta-Binomial prior for the model size. The prior specification for 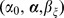 is avoided in BRI, as it directly works on the marginal likelihood 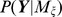 with the parameters 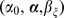 replaced by their MLE. The significance of global association is determined using the Bayes factor calculated in (4) with posterior samples. The rare variants are selected based on the marginal Bayes factor which, for any two variants, is defined as the ratio of the odds of their posterior marginal inclusion probabilities to the odds of their prior marginal inclusion probabilities.

- *Combined multivariate and collapsing (CMC) test* [4]: CMC is a variant pooling method in which the rare variants are grouped according to their allele frequency. After grouping, the rare variants are collapsed into an indicator variable, and then a multivariate test such as Hotelling's 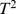 test is applied to the collection formed by the common variants and the collapsed super-variant.
- *Weighed sum Statistic (WSS) test* [5]: WSS is a variant pooling method. It first calculates for each subject a genetic score, which accumulates the rare variants counts within the same gene with a weighting term that emphasizes alleles with a low frequency in controls. Then the scores for all subjects are ordered, and the WSS is computed as the sum of the ranks for the cases. The significance is determined by a permutation procedure.
- *Sequence kernel association (SKAT) test* [6]: SKAT is a random effect model-based method. It assumes a common distribution for the genetic effects of different variants and test for the null hypothesis that the distribution has zero variance.
- *RARECOVER* [9]: RARECOVER is a variable selection-based method. It selects variants in a manner of forward variable selection: Starting from a null model without any genetic variants, the variants are added into the model one by one based on their statistical significance. The significance of global association is determined by a permutation procedure.

The implementation of BRI is available in the R package BVS, the implementation of SKAT is available in the R package SKAT, and the implementations of CMC, WSS, and RARECOVER are available in the R package AssotesteR. In this paper, all the methods are run under their default settings unless otherwise stated.

## Results

### Global Power

**Figure 1:**
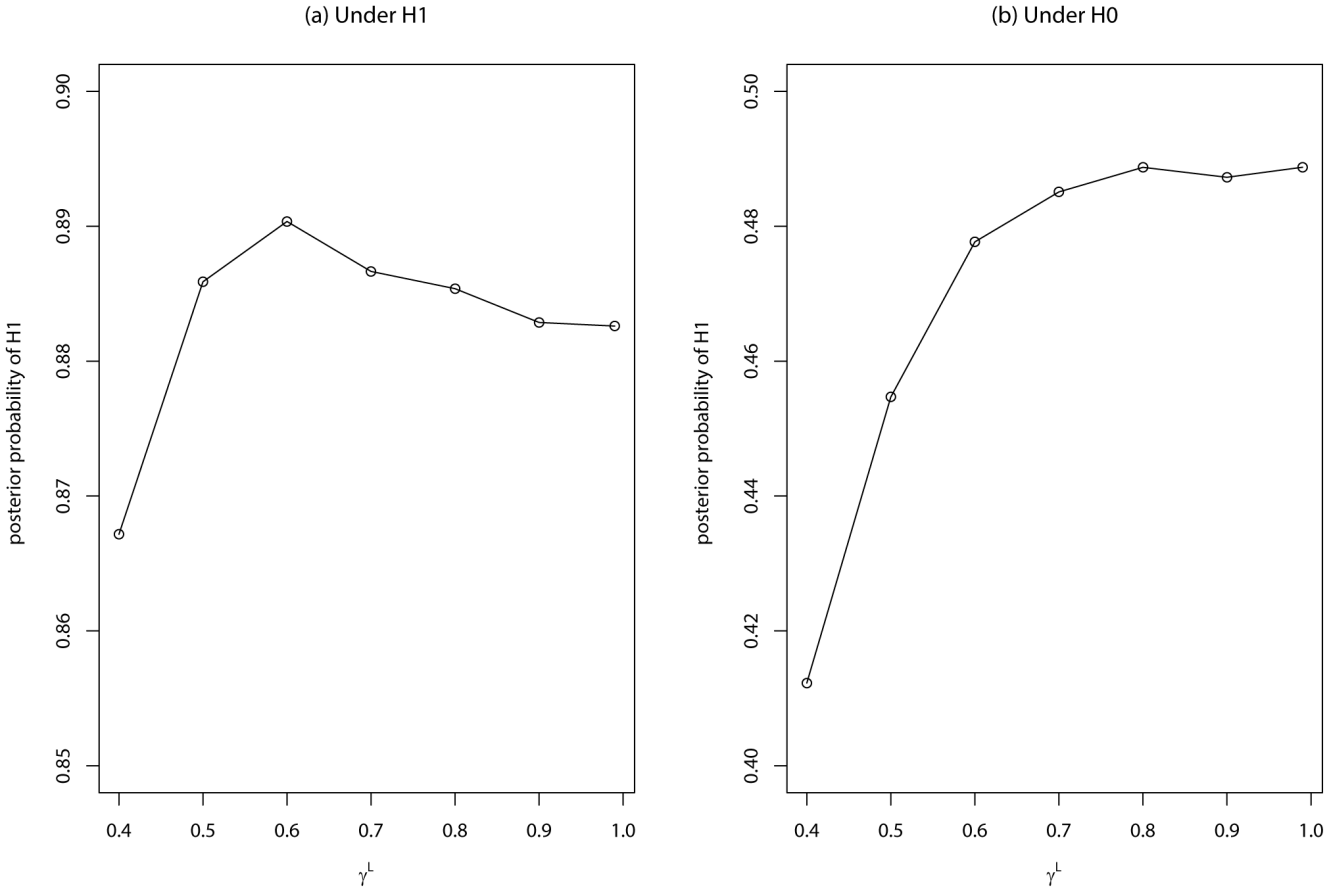
The average posterior probability 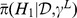 versus 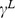 for the datasets simulated under 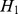 (plot (a)) and under 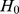 (plot (b)).

We first aim to examine the power of the BRVD versus alternative methods for global association tests. The BRVD has a prior hyperparameter 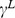 to tune. To determine the value of 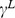, we tried the values 0.4, 0.5, …, 0.9, and 0.99 for all the 200 simulated datasets. For each dataset and each value of 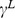, SAMC was run for 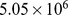 iterations, where the first 50000 iterations were for the burn-in process and the samples generated from the remaining iterations were used for inference. The gain factor sequence was set in (17) with 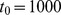, and the sample space 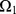 was partitioned into 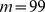 equally spaced (in energy values) subregions with 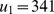 and 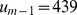. Figure 1 (a) & (b) show the average posterior probability 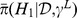 versus 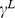 for the datasets simulated under 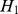 and 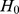, respectively, where the average is calculated over 100 datasets. To indicate the dependency of the average posterior probability on 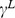, we include 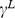 in the notation. For the datasets simulated under 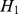, 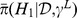 attains its maximum at 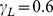; and for the datasets simulated under 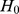, 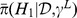 attains its maximum at 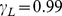. This is interesting: A small value of 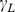 encourages selection of variants, while a large value of 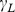 discourages selection of variants. This is consistent with our design of the study: More variants are preferred to be selected for the datasets simulated under 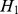. Figure 1 shows 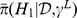 versus different values of 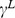: 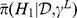 changes only about %2 over the interval 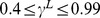 for the datasets simulated under 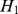, and changes only about 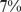 for the datasets simulated under 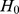. Therefore, we may conclude that the posterior probability 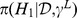 is quite robust to the choice of 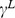.

**Figure 2:**
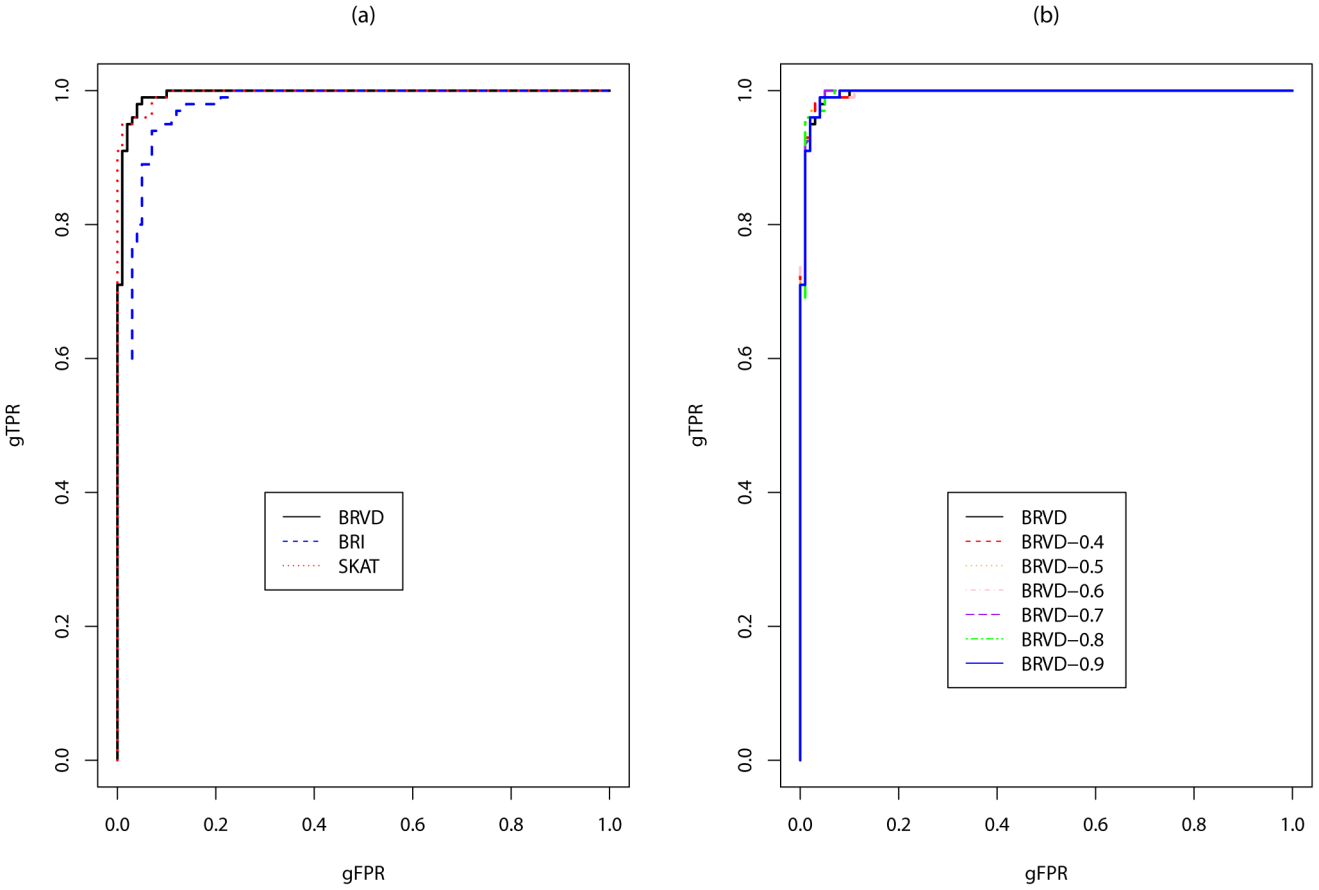
Global ROC curves for BRVD versus BRI and SKAT for the simulated example (with covariate adjustment). Each plot represents a ROC curve as we vary the global BF threshold for BRVD and BRI, and vary the 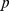-value threshold for SKAT.

Since BRVD, BRI and SKAT are all developed under the regression setting, they are able to adjust for covariates, such as age, gender, race, etc. For this reason, we first compare the powers of these three methods with the simulated covariates adjusted in regression. Figure 2 compares the ROC curves for the global association test, which plots the global false-positive rate (gFPR) versus global true-positive rate (gTPR) as the global BF threshold varies for BRVD and BRI, and the 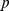-value threshold varies for SKAT. As in BRI, the gFPR is calculated as the ratio of the number of null datasets (the datasets simulated under 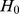) for which a global association has been detected versus the total number of null datasets, and the gTPR is calculated as the number of associated datasets (the datasets simulated under 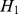) for which a global association has been detected versus the total number of associated datasets. Figure 2(a) shows that for this example, BRVD has about the same power as SKAT and much greater power than BRI to detect a global association. Note that in this plot, we have followed the procedure suggested in Section 2.1 to calculate the gFPR for the null datasets with 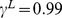 and calculate gTPR for the associated datasets with 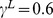. To show the performance of BRVD is robust to the choice of 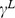, we plot in Figure 2(b) a few ROC curves, where for each curve both gFPR and gTPR were calculated at the same value of 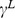. The plot indicates that the BRVD is very robust to the choice of 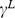 for global association tests.

**Figure 3:**
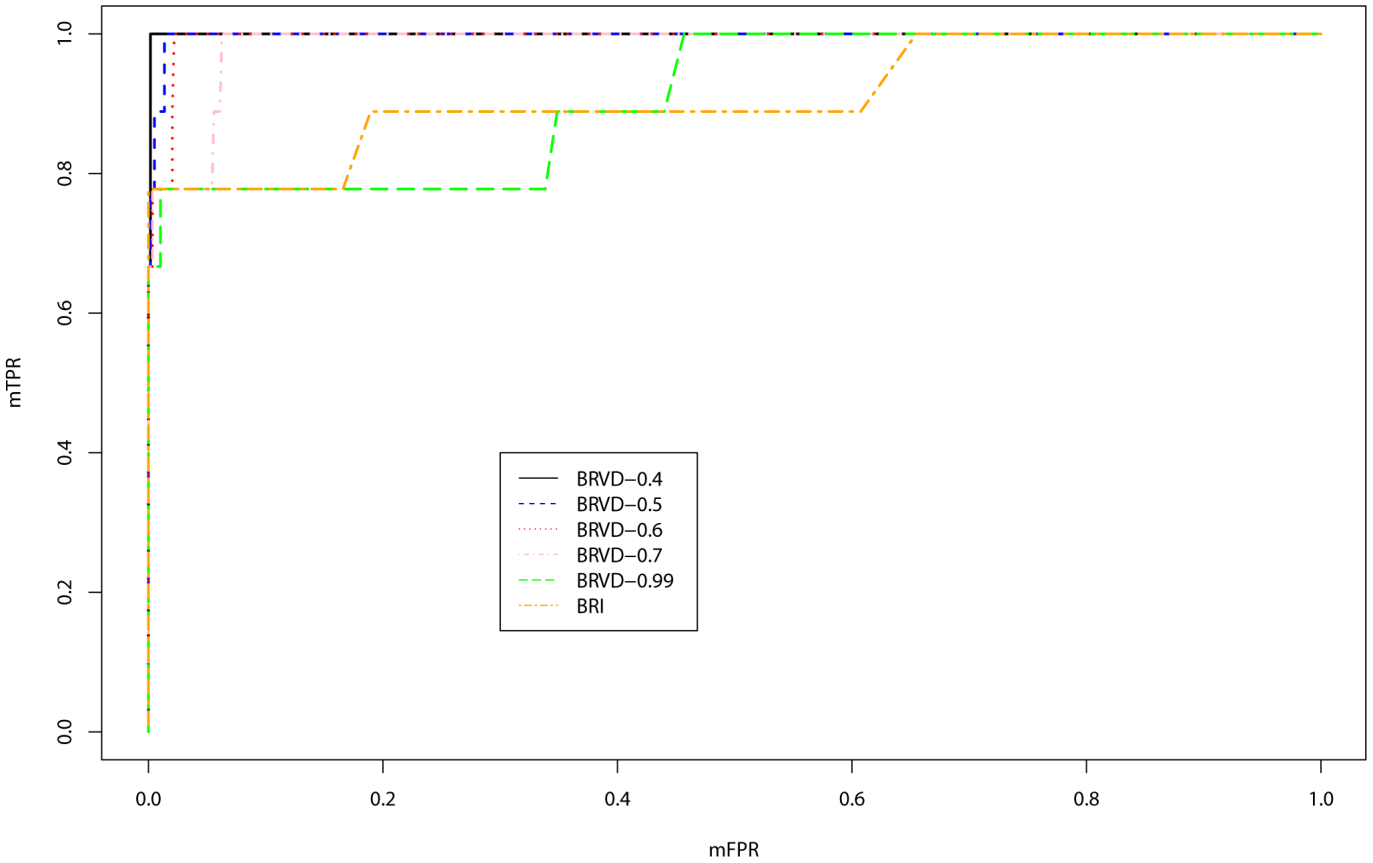
Global ROC curves for BRVD versus BRI, SKAT, CMC, WSS and RARECOVER for the simulated examples (without covariate adjustment). Each plot represents a ROC curve as we vary the global BF threshold for BRVD and BRI, and vary the 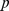-value threshold for SKAT, CMC, WSS and RARECOVER.

The CMC, WSS and RARECOVER cannot be adjusted for covariates. To compare with them, we re-run the BRVD, BRI and SKAT methods on the simulated datasets with the covariates omitted. The effect of covariate omission on test power has been discussed in the literature [29], [30], [31]. The results seem mixed. Under certain situations, such as rare diseases and large sample sizes, omitting the covariates, which are known to affect disease susceptibility and are independent of tested genotypes, can increase the power to detect new genetic associations; whereas, for common diseases, it can decrease the power [31]. For BRVD, SAMC was run for these datasets with the same setting as for the case with covariates adjusted. Figure 3(a) compares the ROC curves of the six methods for global association tests. It shows that when covariates are omitted, BRVD has much greater power than all other methods. Compared to Figure 2(a), we may conclude that BRVD is more robust to covariate omission than the SKAT method. This is important for the success of a method, as in practice we may inevitably have some covariates omitted due to the limitation of our measurements. Figure 3(b) compares the ROC curves of BRVD calculated with different values of 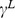. It shows again that the power of BRVD is robust to the choice of 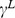 for global association tests.

**Figure 4:**
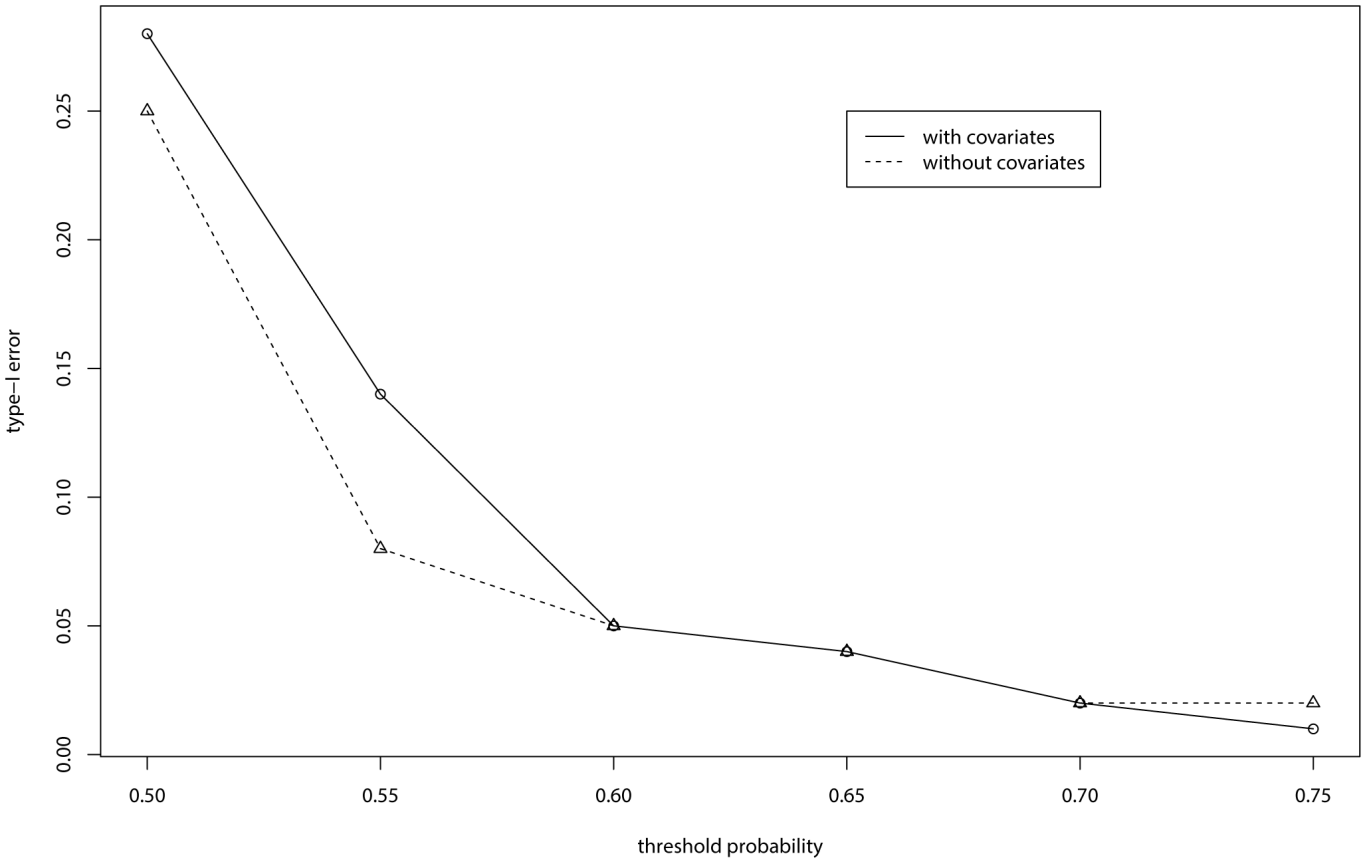
Type-I errors of BRVD for the simulated examples.

In addition to the power, we also explored the type-I error of the global association test based on the testing statistic 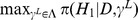 for the simulated examples, where 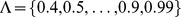 and the prior probabilities 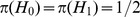. The results, for both cases with and without covariate adjustment, are summarized in Figure 4. Following from Table 1, we suggest to choose 0.75 as the threshold value of 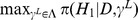; that is, rejecting 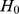 if 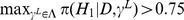. With this threshold value, the resulting type-I errors are 0.01 and 0.02 for the cases with and without covariate adjustment, respectively.

### Rare Variant Detection

**Figure 5:**
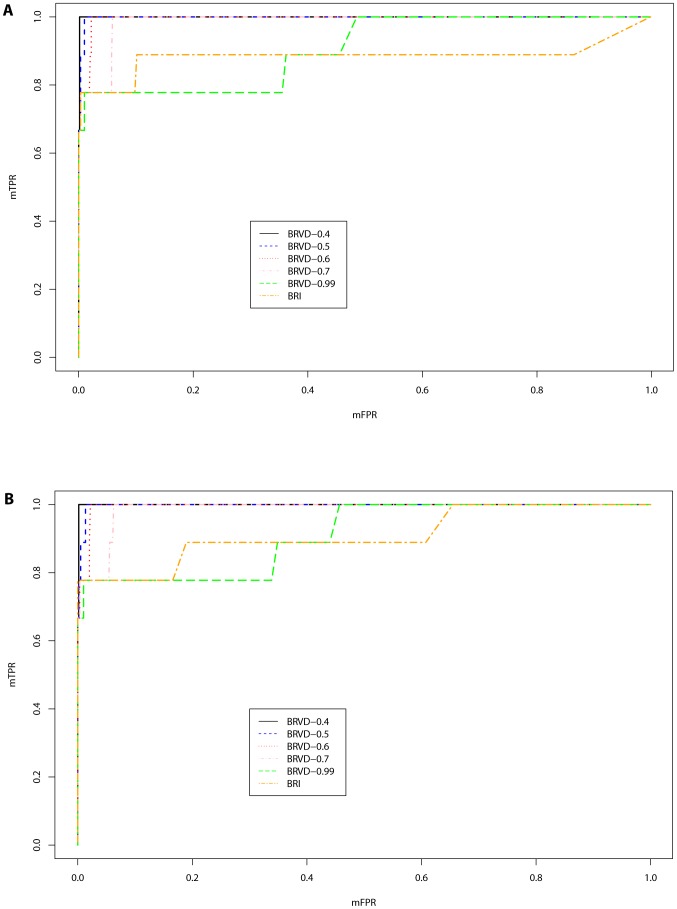
Marginal ROC curves for BRVD and BRI: Left panel: ROC curves with covariates adjusted; right panel: ROC curves with covariates omitted.

Our next aim is to detect rare variants that are associated with the disease, provided that the global association test shows a positive support for the hypothesis 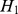. Figure 5 compares the ROC curves of BRVD and BRI for rare variant detection, which are calculated based on the 100 datasets simulated under 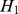. The ROC curves plot the marginal false-positive rate (mFPR) versus marginal true-positive rate (mTPR) as the marginal inclusion probability threshold varies for BRVD and the marginal BF threshold varies for BRI. As in BRI, the mFPR is calculated as the ratio of the number of non-associated variants for which a marginal association has been detected versus the total number of non-associated variants, and the mTPR is calculated as the ratio of the number of associated variants for which a marginal association has been detected versus the total number of associated variants. In drawing Figure 5, the marginal inclusion probabilities for both BRVD and BRI have been averaged over 100 datasets. The left panel of Figure 4 shows the ROC curves for the case with covariates adjusted, and the right panel shows for the case with covariates omitted. In both cases, the BRVD has much greater power than BRI for detection of causal rare variants, especially when 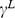 is small, e.g., 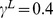, 0.5 and 0.6. When 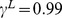, under which all alleles are treated equally, the BRVD has about the same power as BRI. It is worth noting that the BRVD yields its worst result at 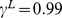.

For global association tests, we suggest to choose the value of 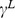 such that the Bayes factor BF(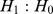) is maximized. Figure 5 suggests that this is still a reasonable rule for determining the value of 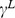 even when our aim is to detect causal rare variants. At 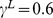, BRVD performs reasonably well: The top 9 variants (ranked in marginal inclusion probabilities) include 7 causal variants, and variants 1 and 2 are ranked 22 and 19, respectively. For this example, we find that a smaller value of 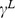 may result in a greater power of BRVD to detect causal rare variants. For example, at 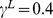, the top 10 variants include all 9 causal variants, and variants 1 and 2 are ranked 4 and 9, respectively. At 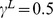, the top 10 variants include 8 causal variants (1,3–9), and variant 2 is ranked 15. This is remarkable, as both variants 1 and 2 have very low MAFs. In BRI, although the variants 3–9 have high ranks in their marginal BFs, variants 1 and 2 are ranked 542 and 68, respectively. This implies that BRI essentially fails to detect variants 1 and 2. The results of this example suggest an alternative rule for determining the value of 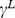: If we aim to detect rare variants, we may choose a small value of 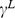 such that some rare variants, such as those singleton variants, can be ranked high in their marginal inclusion probabilities, provided that the association set includes some singleton variants in *a priori* knowledge.

**Figure 6:**
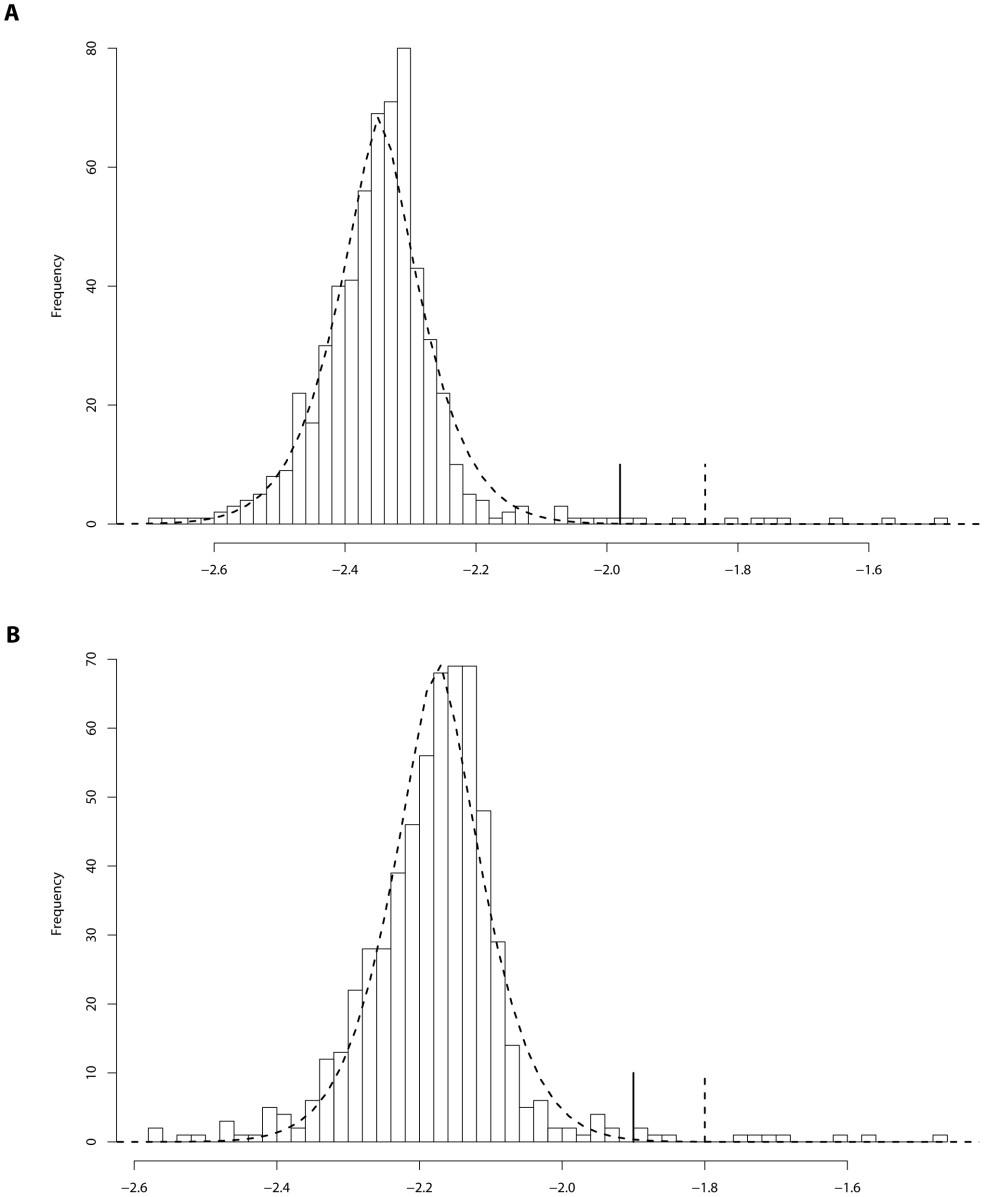
Illustrative plot for causal rare variants detection. The dashed curve shows the fitted density function for the marginal inclusion scores of non-associated variants, and the vertical bar shows the classification rules at the FDR level 0.05 (solid line) and the FDR level 0.01 (dashed line). The left panel is for 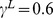 and the right panel is for 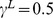.

Figure 6 illustrates how to identify causal variants based on their marginal inclusion scores. The left panel of Figure 6 shows the result for 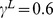. At the FDR level of 0.05, 10 variants are identified as causal variants, and 7 of them (including variants 3–9) are true causal variants. At the FDR level of 0.01, 7 variants are identified and 6 of them (variants 4–9) are true. The right panel of Figure 6 shows the result for 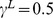. At the FDR level of 0.05, 11 variants are identified as causal variants, and 8 of them (variants 1, 3–9) are true. At the FDR level of 0.01, 7 variants are identified and 6 of them (variants 4–9) are true. The results for other values of 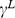 are similar.

### Application to the Early-Onset Myocardial Infarction (EOMI) Exome Sequence Data

The EOMI data (downloaded from dbGaP) is from the NHLBI€s Exome Sequencing Project (ESP), which was designed to identify genetic variants in coding regions (exons) of the human genome that are associated with heart, lung and blood diseases. The dataset consists of 278,263 SNPs in 905 subjects (467 cases and 438 controls) with European origin (EA). After removing the common variants (with MAF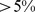) and the variants with zero MAFs, the number of variants is reduced to 113,438. A direct application of BRVD to this dataset is time consuming as it may need an order of 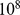 iterations. In addition, the whole dataset need to be scanned once for each iteration. To resolve this issue, we propose, based on the strategy of divide-and-conquer, the following procedure:

Parallel BRVD

(a) (Dividing) Divide the variants into subsets that are of an acceptable size in computation.

(b) (Parallel conquering) Apply BRVD to each of the subsets and identify putative associated variants from the subsets for which the hypothesis 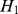 is supported.

(c) (Combining) Combine the variants identified at step (b) into a new dataset, the so-called selected subset data; and then apply BRVD to the selected subset data to identify causal rare variants.

For each subset, the logistic regression model is potentially misspecified because the causal variants located in other subsets are not included in the regression. If some causal variants are missed, we can expect that the BRVD will find some surrogate variants within the subset for the missing causal variants, and the number of surrogate variants can often be greater than the number of missing causal variants. For this reason, we suggest a high FDR level, say, 0.25 or even higher, to be used for identifying putative causal variants from each subset. For the selected subset data, we can expect that it will include the causal variants, surrogate variants of some causal variants, and some noise variants. It is obvious that Lemma 1 and Lemma 2 are still applicable to the selected subset data. By these two lemmas, the parallel BRVD can also select causal variants consistently.

The global association test can also be done on the selected subset of variants. However, a direct application of the BRVD to this subset can lead to a biased test, although for which the power can be very high. This is the same for all other testing procedures. To avoid the bias, a permutation method can be used to evaluate the 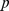-value of the test. For example, one can permute the response variable a large number of times. For each of permuted datasets, the parallel BRVD can be applied to identify a selected subset of variants and then obtain a Bayes factor for the global association test based on the selected subset. Finally, a 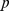-value can be calculated based on the Bayes factors of the permuted datasets.

For the EOMI dataset, we divide the variants into 22 subsets according to the chromosomes where they belong to. The numbers of variants on the 22 chromosomes range from 1,271 (on chromosome 21) to 11,491 (on chromosome 1), which are all acceptable to our current computing facility. BRVD was run 5 times for each subset at each value of 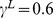, 0.7, 0.8 and 0.9, and each run consisted of 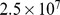 iterations. The gain factor sequence was set in (17) with 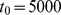, and the sample space 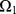 was partitioned into 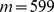 equally spaced (in energy values) subregions with 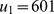 and 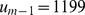. Table 2 summarizes the posterior probabilities of 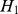 for the 22 chromosomes. The support for the hypothesis 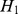 is overwhelming: 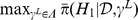 is greater than 0.5 for all 22 chromosomes, where the probability 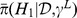 is calculated by averaging over 5 independent runs and 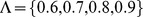 denotes the set of values of 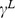 we have tried. According to the value of 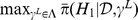, the chromosomes can be classified into two groups: chromosomes 13, 2, 3 and 19 are in the first group with 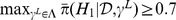, and all other chromosomes are in the second group with 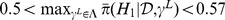. Among the first group chromosomes, chromosomes 13 and 2 provide “substantial” evidence for the global association.

**Table 2:** BRVD results for the EOMI data.

Chromosome	size	*γ^L^*	mean	SD
13	1811	0.9	0.9516	0.0046
2	8383	0.8	0.8059	0.0079
3	6534	0.9	0.7356	0.0080
19	8216	0.9	0.7069	0.0016
other	1271∼12491	—	0.5∼0.57	—

Size: the number of variants included in each chromosome; 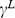: the selected value of 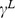; mean: 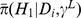, i.e., the average value of 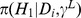 over five independent runs at the selected value of 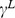; SD: standard deviation of 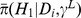.

**Figure 7:**
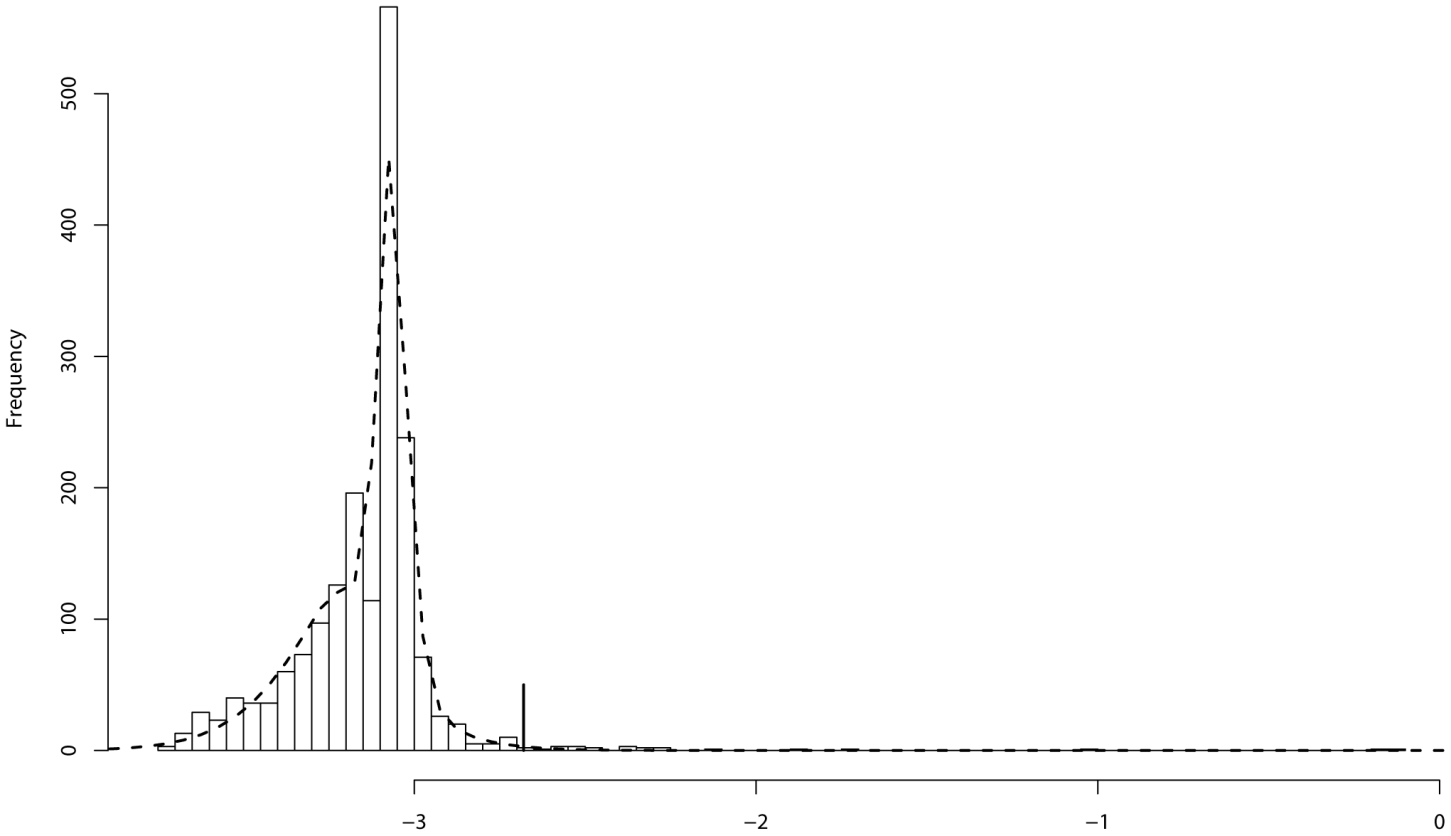
Variant selection from Chromosome 13 for the EOMI data: The dashed curve shows the fitted density function for the marginal inclusion scores of non-associated variants, and the vertical bar shows the classification rules at the FDR level 0.25.

Since all chromosomes show positive support for the global association, putative associated variants should be identified from each of them. For illustration, we here work on the first group chromosomes only. Figure 7 illustrates the selection of putative associated variants from chromosome 13. At a FDR level of 0.25, 24 variants were identified from this chromosome. In the same procedure, 42, 32, and 39 variants were identified from chromosomes 2, 3, and 19, respectively. Putting all the selected variants together form a selected subset of 137 variants.

The BRVD was then applied to the selected subset of variants with the same setting as described above except for sample space partitioning and 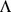. For the selected subset data, 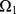 was partitioned into 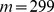 equally spaced (in energy values) subregions with 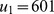 and 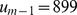, and the values of 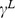 we tried include 0.5, 0.6, …, 0.9. A smaller value of 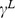 was tried here as 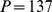 is very small for the selected subset. At each value of 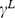, the BRVD shows a decisive support to the hypothesis 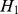 with the estimate of the posterior probability 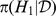 being nearly equal to 1. For example, at 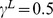, the BRVD produced an estimate of 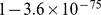 for 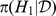. As discussed above, this estimate of 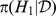 can be biased for the global association test. At 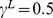, the BRVD identified 10 variants as causal variants at the FDR level 0.1, and identified 14 variants as causal variants at the FDR level 0.2. Table 3 shows the 14 variants in the order (from high to low) of their marginal inclusion probabilities. Among the 14 variants, there are two variants with the MAF lower than 1%. The results for other values of 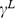 are similar.

**Table 3:** Top 14 variants identified by BRVD for the EOMI data at a FDR level of 0.2.

No.	Variant	Gene	Chrom	MAF		No.	Variant	Gene	Chrom	MAF
1	rs65245292	SLC1A4	2	1.38%		8	rs194325058	TMEM44	3	3.26%
2	rs194408716	FAM43A	3	2.54%		9	rs28827533	PLB1	2	1.05%
3	rs39586979	C13orf23	13	2.76%		10	rs19961331	EFHB	3	4.81%
4	rs39424253	FREM2	13	3.76%		11	rs94197611	GPC6	13	0.99%
5	rs51994587	PCBP4	3	1.33%		12	rs128695828	NO-Gene	3	1.49%
6	rs39424254	FREM2	13	3.76%		13	rs242610172	ATG4B	2	1.55%
7	rs549728	GZMM	19	2.38%		14	rs57867517	ZNF304	19	0.94%

Our method is surprisingly successful for this example: A few rare variants identified by it have been verified in the literature. It is reported that SLC1A4 is associated with atherosclerosis [32], TMEM44 regulates low-density lipoprotein receptor (LDLR) levels which in turn is a critical factor in the regulation of blood cholesterol levels [33], GPC6 is associated with breast cancer [34], and schizophrenia and bipolar [35] and PCBP4 is associated with lung cancer [36].

For comparison, BRI and SKAT were also applied to this example. BRI was run for 50,000 iterations for each of the 22 subsets. The outputs show that only chromosome 2 provides “substantial” evidence for the global association with a Bayes factor of 7.1. The Bayes factors for all other chromosomes are less than 1. On chromosome 2, BRI identified three SNPs, rs65245292, rs179455352 and rs28827533, whose marginal Bayes factor are all greater than 10. It is interesting to point out that both SNPs, rs65245292 and rs28827533, have been identified by BRVD as shown in Table 3. Although the SNP rs179455352 is not included in Table 3, it has been selected by BRVD in the parallel conquering step.

**Figure 8:**
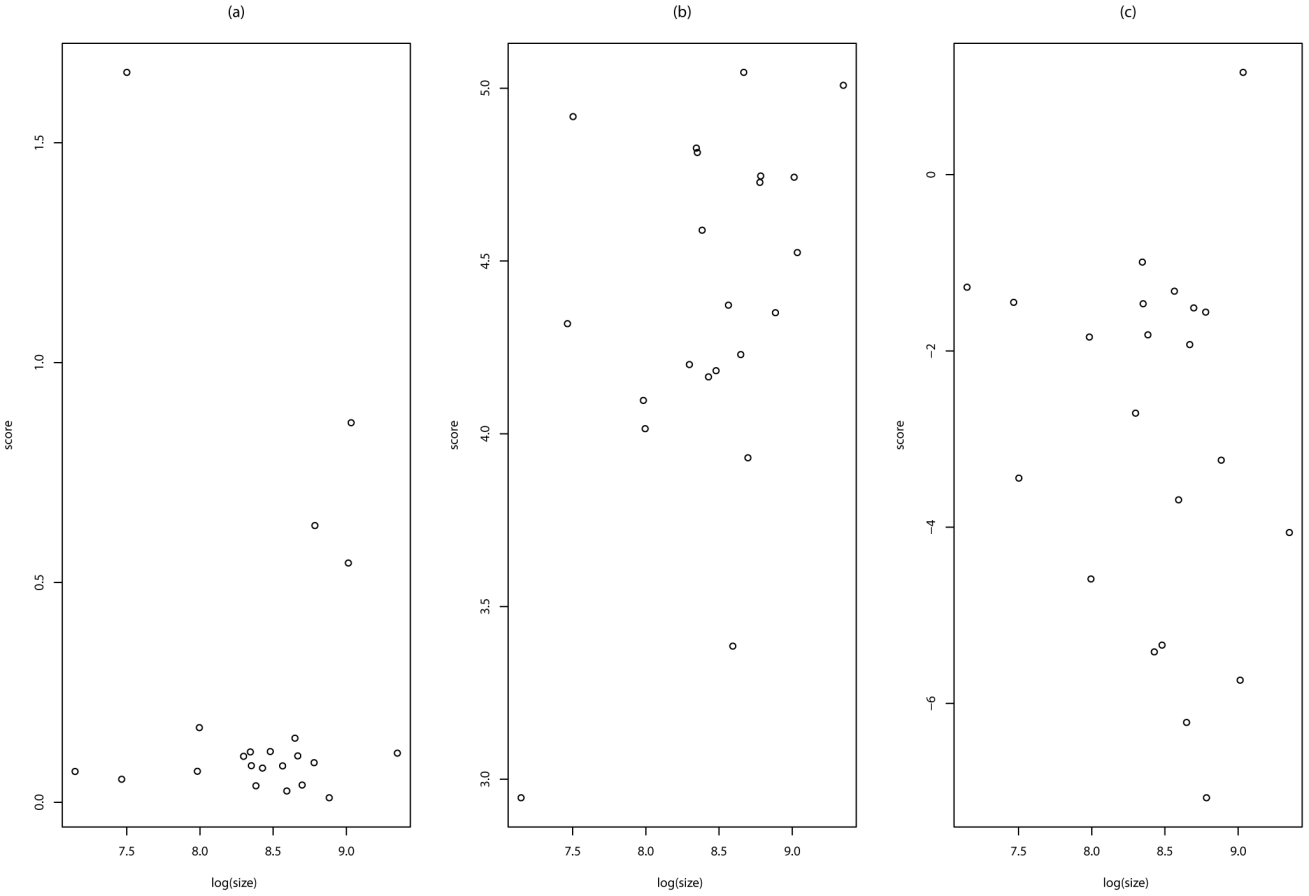
Significance of global association tests versus chromosome length for the EOMI data: (a) BRVD; (b) SKAT; and (c) BRI.

SKAT produced a small 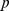-value for each of the 22 subsets, ranging from 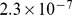 (chromosome 12) to 0.0016 (chromosome 21). According to the 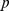-values, all chromosomes are associated with heart, lung and blood diseases. This result suggests that SKAT may be liberal in global association tests. To explore the relationship between the 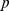-value and the chromosome length, we plot in Figure 8(b) the scatterplot of 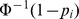 versus 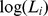, where 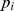 denotes the 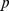-value of chromosome 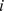, 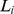 denotes the length of chromosome 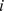, and 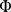 denotes the CDF of the standard normal distribution. The scatterplot indicates that SKAT tends to produce a smaller 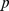-value for a longer chromosome; that is, it tends to be sensitive to the proportion of causal variants.

Similarly, we plot in Figure 8(a) the scatterplot of 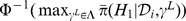 versus 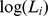 for BRVD, where 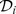 denotes the subset corresponding to chromosome 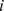; and plot in Figure 8(c) the scatterplot of 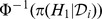 versus 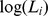 for BRI, where 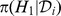 is calculated from the Bayesian factor with the prior probabilities 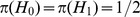. Although BRI is not as sensitive to the chromosome length as SKAT, its results suggest that it is pretty conservatives in global association tests. As discussed above, the literature results show that chromosome 3 and chromosome 13 are also associated with heart, lung and blood diseases, but BRI failed to identify these associations. In summary, the comparison implies that BRVD outperforms both SKAT and BRI for this real-data example.

### Computational time

**Figure 9:**
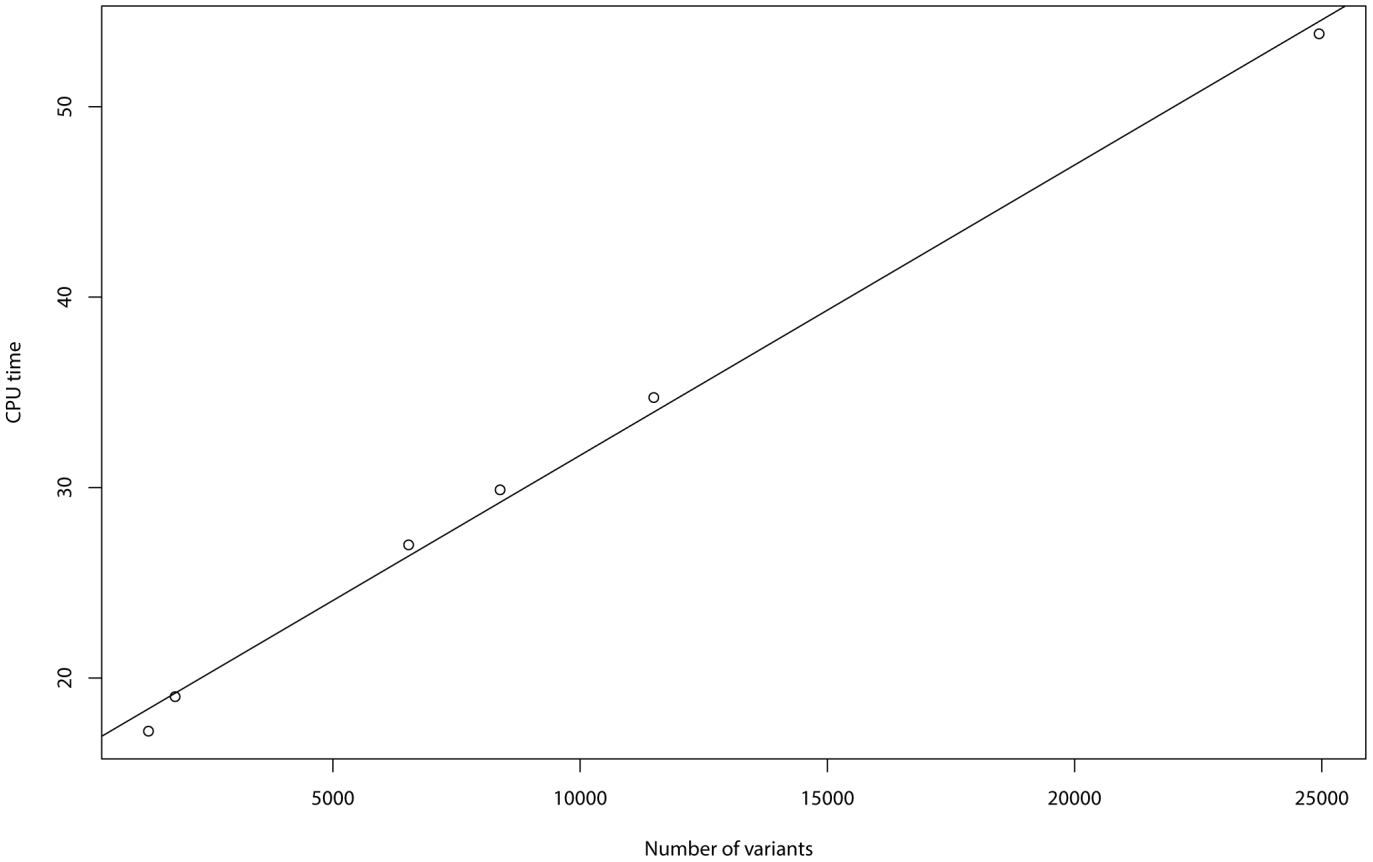
The CPU time of BRVD versus the number of variants for the EOMI data (with 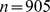).

The computation time for the BRVD depends on the sample size (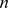) and the number of variants (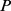). Table 4 recorded the CPU time cost by BRVD on an Intel Xeon E5-2690 processor for running 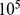 iterations under different settings of 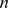 and 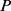. A linear regression analysis of the CPU time versus 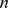 and 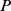 produces a 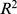 of 99.76%, which indicates an adequate fitting of the regression. Both 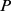 and 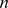 are significant for the regression, and their 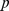-values are 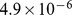 and 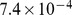, respectively. Figure 9 plots the CPU time of BRVD versus 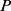 for the EOMI data (with 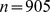). It indicates a strong linear relationship between the CPU time and 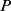. Since the number of iterations is usually set to be proportional to the value of 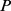, this analysis implies that the CPU time of the BRVD can increase as a quadratic function of 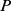.

**Table 4:** CPU time cost by the BRVD on an Intel Xeon E5-2690 processor (2.9 GHz) for running 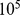 iterations.

Case	*n*	*P*	CPU(s)
1	500	600	7.67
2	905	1,271	17.21
3	905	1,811	19.02
4	905	6,534	26.99
5	905	8,383	29.88
6	905	11,491	34.73
7	905	24,944	53.82

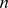
: sample size; 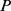: number of variants.

In analyzing the CPU time of BRVD, we fixed 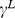 to 0.9. We note that the CPU time of BRVD can slightly increase as 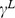 decreases for fixed values of 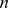 and 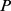, because a smaller value of 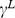 tends to result in a larger model. However, the effect of 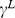 is not significant, because, under the control of multiplicity, the sizes of the selected models are always tiny compared to the value of 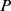. The CPU time of the BRVD is dominated by the part of data scanning that needs to be performed for each iteration.

## Discussion

In this paper, we have developed a new Bayesian method, the so-called BRVD, for detection of causal variants. The BRVD simultaneously addresses two issues: (i) Are there any of the variants associated with the disease, and (ii) Which variants, if any, are driving the association. The BRVD is developed based on the theory of posterior consistency, under which the causal variants can be identified consistently. The numerical results indicate that the BRVD is more powerful for global association tests than the existing methods, such as CMC, WSS, SKAT, C-alpha, RARECOVER, VT, and BRI, and also more powerful for detection of causal variants than the BRI method. In this paper, we have also developed a parallel version of BRVD based on the strategy of divide-and-conquer. The parallel BRVD can be conveniently used for the datasets for which the number of variants is extremely large.

Since the BRVD is developed under the framework of logistic regression, it can be directly applied to identify gene-gene and gene-environment interactions by including in the model some interaction terms of SNP-SNP and SNP-covariates. A gene-gene and/or gene-environment interaction network can then be constructed. This method is very flexible, depending on the specification of interaction terms. For example, to explore complex higher-order interactions, a partially linear tree-based regression model [37] may be used.

Although BRVD has a high power for both the global association tests and causal variants detection, its power can be further improved by employing a more sophisticated weighting scheme for the variants. The current weighting scheme depends on the MAF only. In the future, one may incorporate other biological information, e.g., the gene information, into the weighting scheme. This may help further to identify the causal variants whose MAFs are extremely low. In the current implementation of the BRVD, the SAMC algorithm is used for sampling from the posterior. At each iteration, a variant is randomly selected to undergo a model update of variant addition, deletion, or exchange. In the future, a SAMC algorithm with an adaptive proposal may be used. The new version of SAMC allows one to select a variant for model update based on the working estimate of marginal inclusion probabilities. In the limit case, the new version of SAMC will update the model according to the marginal inclusion probabilities of all variants. Therefore, it can converge faster than the standard version of SAMC.

For global association tests, the BRVD can also be used in conjunction with other frequentist methods, such as SKAT, if one is interested in a 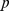-value measurement for the significance of the test. One can first apply the BRVD to select a subset of variants and then conduct the association test on the selected subset of variants using the frequentist method. Since all the existing rare variant testing methods seem to be sensitive to the proportion of causal variants [38], the combined use of the BRVD and frequentist methods can generally reduce the sensitivity of the test methods to the proportion of causal variants.

The BRVD is general in the sense that it can be used for rare variants, common variants, and also a joint analysis of common and rare variants. In the case of joint analysis, its power for detecting rare variants will not be affected much if 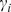 in (9) is chosen appropriately as an increasing function of MAF. We note that in the literature some other Bayesian variable selection methods have also been developed and can potentially be used for variant selection [39], [40], [41]. However, none of these methods is directly comparable with BRVD. The method [39] is developed for linear regression under the framework of large-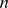-small-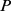, and thus cannot be applied to the small-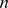-large-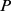 logistic regression problems considered in this paper. The method [40] is developed for linear regression, although for the small-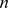-large-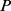 problems; hence, it cannot be compared with BRVD for logistic regression. The method [41] aims to identify biomarkers, for which the model incorporates the biological information on known pathways and gene-gene networks. Since these information are not available for the problems considered in this paper, this method cannot be directly compared with BRVD. Also, we note that although BRVD and the methods [40], [41] are all applicable to the small-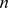-large-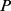 problems, BRVD has a theoretical advantage over the other two methods: BRVD is consistent, i.e., the causal variables can be identified by it in probability 1 as the sample size 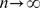; while this is unclear for the other two methods.

In this paper, BRVD is developed for dichotomous phenotypes only. The framework of BRVD can be easily extended to continuous phenotypes. For continuous phenotypes, linear regression can be used to relate the phenotype to the variants, and appropriate prior distributions that lead to the posterior consistency need to be specified for the model and model specific parameters. Alternatively, one can impose a non-local prior on the model parameters as in [42]. Under the non-local prior, it can be shown that the causal variants can be consistently identified if the total number of variants is bounded by the number of subjects.

## Supporting Information

File S1 **Supporting Information.**
(PDF)
