# Supplementary material for: Bayesian Detection of Causal Rare Variants under Posterior Consistency
Source: PLoS One. 2013 Jul 26;8(7):e69633. doi: 10.1371/journal.pone.0069633 (PMC3724943; doi:10.1371/journal.pone.0069633)
Supplement: File S1 — Supporting Information. (PDF) [file pone.0069633.s001.pdf]

Supporting Document for "Bayesian Detection of Causal Rare Variants under Posterior Consistency"

Faming Liang*[1], Momiao Xiong[2]
1 Department of Statistics, Texas A&M University, College Station, TX 77843, USA
2 Division of Biostatistics, University of Texas School of Public Health, Houston, TX 77030, USA
∗ E-mail: fliang@stat.tamu.edu

S0: The Posterior Distribution

Let σ^2 denote the common prior variance of α_i and β_i^ξ. Under the assumption of prior independence, we have

$$\begin{aligned}\pi(\alpha_0,&\boldsymbol{\alpha}, M_\xi, \boldsymbol{\beta}_\xi, \sigma^2, \mathcal{D}|H_1) = \pi(\alpha_0, \boldsymbol{\alpha}, M_\xi, \boldsymbol{\beta}_\xi, \sigma^2|H_1, \mathcal{D})\pi(\mathcal{D}|H_1) \\ &= f_1(\boldsymbol{Y}|\alpha_0, \boldsymbol{\alpha}, M_\xi, \boldsymbol{\beta}_\xi)\pi(\alpha_0, \boldsymbol{\alpha}|\sigma^2)\pi(M_\xi|H_1)\pi(\boldsymbol{\beta}_\xi|M_\xi, H_1, \sigma^2)\pi(\sigma^2) \\ &= \prod_{i=1}^n \left\{[P(Y_i=1|\alpha_0, \boldsymbol{\alpha}, M_\xi, \boldsymbol{\beta}_\xi, H_1)]^{Y_i}[P(Y_i=0|\alpha_0, \boldsymbol{\alpha}, M_\xi, \boldsymbol{\beta}_\xi, H_1)]^{1-Y_i}\right\} \prod_{i=0}^q \left[\frac{1}{\sqrt{2\pi}\sigma} e^{-\alpha_i^2/2\sigma^2}\right] \\ &\times \prod_{i=0}^{|\xi|} \left[\frac{1}{\sqrt{2\pi}\sigma} e^{-(\beta_i^\xi)^2/2\sigma^2}\right] \frac{b^a}{\Gamma(a)[\Gamma(a, b/A) - \Gamma(a, b/B)]} \frac{e^{-b/\sigma^2}}{\sigma^{2(a+1)}} \frac{\prod_{i=1}^p \nu_i^{\delta_\xi(i)}(1-\nu_i)^{1-\delta_\xi(i)}}{1-\prod_{i=1}^P (1-\nu_i)},\end{aligned} \tag{1}$$

where $\pi(\alpha_0, \boldsymbol{\alpha}, M_\xi, \boldsymbol{\beta}_\xi, \sigma^2|H_1, \mathcal{D})$ is the posterior distribution of $(\alpha_0, \boldsymbol{\alpha}, M_\xi, \boldsymbol{\beta}_\xi, \sigma^2)$ conditioned on H_1, and $P(Y_i = 1|\cdots)$ denotes the disease probability of subject i under model M_ξ. After integrating out σ^2 over the interval $[A, B]$, we have

$$\begin{aligned}\pi(\alpha_0,&\boldsymbol{\alpha}, M_\xi, \boldsymbol{\beta}_\xi, \mathcal{D}|H_1) = \prod_{i=1}^n \left\{[P(Y_i=1|\alpha_0, \boldsymbol{\alpha}, M_\xi, \boldsymbol{\beta}_\xi, H_1)]^{Y_i}[P(Y_i=0|\alpha_0, \boldsymbol{\alpha}, M_\xi, \boldsymbol{\beta}_\xi, H_1)]^{1-Y_i}\right\} \\ &\times (2\pi)^{-\frac{1+q+|\xi|}{2}} \frac{b^a}{\Gamma(a)} \frac{\Gamma(a+\frac{1+q+|\xi|}{2})}{\left[b+\frac{1}{2}\sum_{i=0}^q \alpha_i^2 + \frac{1}{2}\sum_{i=1}^{|\xi|}(\beta_i^\xi)^2\right]^{a+\frac{1+q+|\xi|}{2}}} \frac{\prod_{i=1}^p \nu_i^{\delta_\xi(i)}(1-\nu_i)^{1-\delta_\xi(i)}}{1-\prod_{i=1}^P (1-\nu_i)} \\ &\times \frac{Q\left(a+\frac{1+q+|\xi|}{2}, \frac{b+\frac{1}{2}\sum_{i=0}^q \alpha_i^2 + \frac{1}{2}\sum_{i=1}^{|\xi|}(\beta_i^\xi)^2}{A}\right) - Q\left(a+\frac{1+q+|\xi|}{2}, \frac{b+\frac{1}{2}\sum_{i=0}^q \alpha_i^2 + \frac{1}{2}\sum_{i=1}^{|\xi|}(\beta_i^\xi)^2}{B}\right)}{Q(a, \frac{b}{A}) - Q(a, \frac{b}{B})}.\end{aligned} \tag{2}$$

Similarly, conditioned on the null hypothesis H_0, we have

$$\begin{aligned}\pi(\alpha_0,&\boldsymbol{\alpha}, \mathcal{D}|H_0) = \pi(\alpha_0, \boldsymbol{\alpha}|\mathcal{D}, H_0)\pi(\mathcal{D}|H_0) \\ &= \prod_{i=1}^n \left\{[P(Y_i=1|\alpha_0, \boldsymbol{\alpha}, H_0)]^{Y_i}[P(Y_i=0|\alpha_0, \boldsymbol{\alpha}, H_0)]^{1-Y_i}\right\} (2\pi)^{-\frac{1+q}{2}} \frac{b^a}{\Gamma(a)} \\ &\times \frac{\Gamma(a+\frac{1+q}{2})}{\left[b+\frac{1}{2}\sum_{i=0}^q \alpha_i^2\right]^{a+\frac{1+q}{2}}} \frac{Q\left(a+\frac{1+q}{2}, \frac{b+\frac{1}{2}\sum_{i=0}^q \alpha_i^2}{A}\right) - Q\left(a+\frac{1+q}{2}, \frac{b+\frac{1}{2}\sum_{i=0}^q \alpha_i^2}{B}\right)}{Q(a, \frac{b}{A}) - Q(a, \frac{b}{B})},\end{aligned} \tag{3}$$

where $\pi(\alpha_0, \boldsymbol{\alpha}|\mathcal{D}, H_0)$ is the posterior distribution of $(\alpha_0, \boldsymbol{\alpha})$ conditioned on H_0, and $P(Y_i = 1|\alpha_0, \boldsymbol{\alpha})$ denotes the disease probability of subject i under the null model.

S1: Consistency of Rare Variant Selection

To justify the BRVD, we refer to the Bayesian theory developed in [1]. Rewrite the dataset as $(y^{(i)}, x^{(i)})_{i=1}^n$ by ignoring the covariates, where $x^{(i)} = (x_1^{(i)}, \ldots, x_{P_n}^{(i)})$ denotes the genotype of subject i. To emphasize the fact that the number of variants in the dataset can increase with the sample size n, we re-denote P by P_n in the Appendix. Let $f = f(y|x)$ denote the true density of y conditioned on x, and let $\hat{f} = \hat{f}(y|x, M_\xi)$ denote the conditional density proposed by the posterior $\pi(M_\xi|\mathcal{D})$. Let $v_x(dx)$ denote the probability measure for x, and $v_y(dy)$ the dominating measure for the conditional densities f and $\hat{f}$. Assume that the data for n subjects are independent and identically distributed based on $f(y,x)v_x(dx)v_y(dy)$. Let

$$d(\hat{f}, f) = \sqrt{\int\int(\sqrt{\hat{f}} - \sqrt{f})^2 v_y(dy)v_x(dx)}$$

denote the Hellinger distance between $\hat{f}$ and f. Furthermore, we assume the following conditions hold:

(A_1) $P_n \succ n^\delta$ for $\delta > 0$, where $b_n \succ a_n$ means $\lim_{n\to\infty} a_n/b_n = 0$.

(A_2) The true model is sparse and satisfies the condition $\lim_{n\to\infty} \sum_{i=1}^{P_n} |\beta_j| < \infty$.

Following Theorem 2 of [1], we have the following lemma:

Lemma 1 (Posterior Consistency) *Consider the logistic regression model specified by (1) of the main text. Assume that the model satisfies the conditions (A_1) and (A_2), and $|x_j| \le 1$ for all $j = 1, \ldots, P_n$. Let ϵ_n be a sequence such that $\epsilon_n \in (0,1]$ for each n and $n\epsilon_n^2 \succ \log(P_n)$. Suppose that the priors for the logistic regression are specified by (5)–(9) of the main text, and that γ_i's and K_n are chosen such that the following conditions hold:*

(B_1) $P_n \le e^{C_1 n^\alpha}$ for some constants $C_1 > 0$ and $\alpha \in (0,1)$ for all large enough n;

(B_2) $\Delta(r_n) = \inf_{\xi_n:|\xi_n|=r_n} \sum_{j:j\notin\xi_n} |\beta_j| \le e^{-C_2 r_n}$ for some constant $C_2 > 0$, where $r_n = \lceil\sum_{i=1}^{P_n} \nu_i\rceil$ denotes the up-rounded expectation of the prior (8) (of the main text);

(B_3) $C_2^{-1}\log(n) \le r_n \le K_n \prec n^\beta$ for some $\beta \in (0,q)$, where $q = \min(1-\alpha, \delta)$.

Then for some $c > 0$ and for all sufficiently large n,

$$P\left\{\pi[d(\hat{f}, f) > \epsilon_n|\mathcal{D}] \ge e^{-0.5cn\epsilon_n^2}\right\} \le e^{-0.5cn\epsilon_n^2}, \quad (4)$$

where $P\{\cdot\}$ denotes the probability measure for the data.

This lemma can be viewed as a corollary of Theorem 2 of [1] for our particular choice of priors. The proof is straightforward as the consequence of the following facts: As implied by (6) (of the main text), the eigenvalues of the covariance matrix of the prior (7) (of the main text) are bounded by the constants A and B, and thus $\max\{\sigma_\beta^2, \sigma_\beta^{-2}\}$ can be bounded by BK_n for some constant $B > 0$.

If we further assume that $P_n = O(n^\kappa)$ for some $\kappa > 0$, then $n^{\kappa(1-\gamma^R)} \prec r_n \prec n^{\kappa(1-\gamma^L)}$. Let $K_n = C_3 r_n$ for some constant $C_3 > 1$. Then α can be set to a number close to zero. If $\kappa > 1$, we can set $\delta = 1$ and thus any $\gamma^L \in (1 - 1/\kappa, 1)$ ensures the conditions (B_1)–(B_3) to be satisfied. If $\kappa \le 1$, we can set $\delta = \eta\kappa$ for any $0 < \eta < 1$ such that $\eta\kappa < 1 - \alpha$, then the choice of any $\gamma^L \in (1-\eta, 1)$ ensures the conditions (B_1)–(B_3) to be satisfied. Pushing α to its limit 0 and η to its limit 1, we have the range $\gamma^L \in (0,1)$ for $\kappa \le 1$. Given the choice of γ^L, the convergence rate can be taken as

$$\epsilon_n \sim n^{-(1-\alpha-\zeta)/2},$$

for some $\zeta \in (\kappa(1-\gamma^L), q)$ with q as defined in (B_3).

To establish the consistency of the variant selection rule $\widehat{\xi}_{\hat{q}}$, we specify the following condition on the identifiability of the true model M_{ξ_*}, where ξ_* denotes the set of true causal variants. Let $A_{\epsilon_n} = \{\xi : d(\hat{f}(y|x, M_\xi), f(y|x)) \le \epsilon_n\}$, where $d(\cdot,\cdot)$ denotes the Hellinger distance between $\hat{f}$ and f. Define

$$\rho_j(\epsilon_n) = \sum_{\xi \in A_{\epsilon_n}} |\delta_{\xi_*}(j) - \delta_\xi(j)| \pi(\xi|\mathcal{D}),$$

which measures the distance between the true model and the sampled models for feature j in the ϵ_n-neighborhood A_{ϵ_n}.

(C_1) (Identifiability of ξ_*) $\max_{j\in\{1,2,\ldots,P_n\}} \rho_j(\epsilon_n) \to 0$ as $n \to \infty$ and $\epsilon_n \to 0$.

This condition states that as $n \to \infty$ and $\epsilon_n \to 0$, the true model is identifiable. In other words, when n is sufficiently large, if a model results in the same density of y as the true density, then the model must coincide with the true model. Following from Theorem 3.1 of [2], we have the following lemma:

Lemma 2 *Assume that the conditions of Lemma 1 and the condition (C_1) hold.*

(i) For any $\epsilon'_n > 0$ and all sufficiently large n,

$$P\left(\max_{1\le j\le P_n} |q_j - \delta_{\xi_*}(j)| \ge 2\sqrt{\epsilon'_n + e^{-0.5cn\epsilon_n^2}}\right) \le P_n e^{-0.5cn\epsilon_n^2}.$$

(ii) (Sure screening) For all sufficiently large n,

$$P(\xi_* \subset \widehat{\xi}_{\hat{q}}) \ge 1 - s_n e^{-0.5cn\epsilon_n^2},$$

where s_n denotes the size of ξ_*, for some choice of $\hat{q} \in (0,1)$, preferably one not close to 0 or 1.

(iii) (Consistency) For all sufficiently large n,

$$P(\xi_* = \widehat{\xi}_{0.5}) \ge 1 - P_n e^{-0.5cn\epsilon_n^2}.$$

The proof of Lemma 2 can be found in [2]. This lemma implies that the posterior probability of the true model will converge to 1, i.e.,

$$\pi(M_{\xi_*}|\mathcal{D}) \to 1, \tag{5}$$

as the sample size n goes to infinity. This is the so-called global model consistency in Bayesian variable selection [3].

S2: The Proposal Distribution Used in SAMC Simulations

The proposal distribution $T(\omega^{(t)}, \omega')$ used in the SAMC algorithm includes four types of moves, variant birth, variant death, variant exchange, and coefficient updating, which are described as follows.

Let M_{ξ_t} denote the model simulated at iteration t, let $\omega^{(t)}$ denote the parameter vector of M_{ξ_t}, and let ξ_t^c denote the set of variants excluded from the model M_{ξ_t}. In the birth step, a variant, say $\boldsymbol{x}_i$, is randomly selected from the set ξ_t^c and then a new model $M_{\xi'}$ is formed by including $\boldsymbol{x}_i$ into the current

model. The regression coefficient for the newly added variant is generated from a normal distribution with mean 0 and variance given by

$$\sigma^2_{\xi_t} = [0.5\sum_{i=1}^{|\xi_t|}(\beta_i^{\xi_t})^2 + b]/[0.5 * |\xi_t| + a], \tag{6}$$

where a and b are prior hyperparameter specified in (6) (of the main text). In the death step, a predictor, say $\boldsymbol{x}_j$, is randomly selected from the set ξ_t and then a new model is formed by removing $\boldsymbol{x}_j$ from the current model. In the exchange step, a predictor, say $\boldsymbol{x}_i$, is randomly selected from the set ξ_t^c and another predictor, say $\boldsymbol{x}_j$, is randomly selected from the set ξ_t, and then a new model $M_{\xi'}$ is formed by replacing $\boldsymbol{x}_j$ by $\boldsymbol{x}_i$ in the current model. The regression coefficient for the variant $\boldsymbol{x}_i$ is generated from a normal distribution with mean 0 and variance as given in (6). The coefficient updating step consists of two substeps, which are to update the covariate coefficients $(\alpha_0, \boldsymbol{\alpha})$ and the variant coefficients $\boldsymbol{\beta}_\xi$, respectively. The probabilities for these two substeps are 0.3 and 0.7, respectively. The hit-and-run algorithm [4] is employed for updating both types of regression coefficients.

Since the death and exchange moves cannot be performed for the null model, and the birth move cannot be performed for the maximum size models, we specify the following proposal probabilities for the four types of moves conditioned on value of $|\xi_t|$:

$$\begin{cases} P(\text{variant birth}\big||\xi_t| = 0) = 1/4, \\ P(\text{coefficient updating}\big||\xi_t| = 0) = 3/4, \\ P(\text{variant birth}\big|0 < |\xi_t| < K_n) = P(\text{variant death}\big|0 < |\xi_t| < K_n) = (\text{variant exchange}\big|0 < |\xi_t| < K_n) \\ \qquad = P(\text{coefficient updating}) = 1/4, \\ P(\text{variant death}\big||\xi_t| = K_n) = P(\text{variant exchange}\big||\xi_t| = K_n) = 1/4, \\ P(\text{coefficient updating}\big||\xi_t| = K_n) = 1/2, \end{cases} \tag{7}$$

where $K_n < P_n$ denotes the maximum model size considered by the user.

S3: Rejection region determination for multiple hypothesis tests

Let $\hat{q}_1, \hat{q}_2, \ldots, \hat{q}_P$ denote the estimates of the marginal inclusion probabilities of the P variants. Let

$$z_i = \Phi^{-1}(\hat{q}_i), \quad i = 1, \ldots, P,$$

denote the corresponding marginal inclusion scores (MIS), where $\Phi(\cdot)$ denotes the cumulative distribution function (CDF) of the standard Gaussian distribution. To identify the variants that have significantly high MIS, we model the MIS by a k-component mixture exponential power distribution, for which the most right component corresponds to the causal variants and the other components correspond to the noise variants. In this paper, k is often set to 2 or 3. For a k-component mixture exponential power distribution, the density function is given by

$$g(z|\vartheta_k) = \sum_{i=1}^{k} \varpi_i \psi(z|\mu_i, \sigma_i, \alpha_i), \tag{8}$$

where $\vartheta_k = (\varpi_1, \mu_1, \sigma_1, \alpha_1, \ldots, \varpi_k, \mu_k, \sigma_k, \alpha_k)$ contains all parameters of the distribution, ϖ_i is the weight of the ith component with $0 < \varpi_i < 1$ and $\sum_{i=1}^m \varpi_i = 1$, and

$$\psi(z|\mu_i, \sigma_i, \alpha_i) = \frac{\alpha_i}{2\sigma_i\Gamma(1/\alpha_i)} \exp\left\{-(|z-\mu_i|/\sigma_i)^{\alpha_i}\right\}, \quad -\infty < \mu_i < \infty,\ \sigma_i > 0,\ \alpha_i > 1, \tag{9}$$

where the parameters μ_i, σ_i and α_i represent the center, dispersion and decay rate of the distribution, respectively. For $\alpha_i = 2$, the distribution (9) is reduced to $N(\mu_i, \sigma_i^2/2)$; for $1 < \alpha_i < 2$, the distribution is heavy-tailed; and for $\alpha_i > 2$, the distribution is light-tailed. The identifiability of (8) has been established in [5].

The parameters ϑ_k can be estimated as in [6] by minimizing the Kullback-Leibler divergence

$$\mathrm{KL}(g_{\vartheta_k}, g) = -\int \log\left\{\frac{g(z|\vartheta_m)}{g(z)}\right\} g(z)dz,$$

where $g(z)$ denotes the unknown true density of z_i's. For a given value of k, the minimization can be done using the stochastic approximation algorithm, refer to [6] for the details. One significant advantage of this algorithm is that it permits the general dependence between z_i's. A proof of convergence for this algorithm can be found in [7]. The cutoff value z_r, which corresponds to the setting $\hat{q} = \Phi(z_r)$, can be chosen by controlling the false discovery rate (FDR) of causal features at a pre-specified test level. For a given rule $\Lambda_r = \{Z_i \geq z_r\}$, the FDR can be estimated by

$$\mathrm{FDR}(\Lambda_r) = \frac{P\sum_{i=1}^{k-1} \hat{\varpi}_i[1 - F(z_r|\hat{\mu}_i, \hat{\sigma}_i, \hat{\alpha}_i)]}{\#\{z_i : z_i \geq z_r\}}, \tag{10}$$

where $\#\{z_i : z_i \geq z_r\}$ denotes the number of features with the MIS greater than z_r, and $F(\cdot)$ denotes the CDF of the exponential power distribution (9). Define the q-value [8] as

$$q_r^s(z) = \inf_{\{\Lambda_r : z \in \Lambda_r\}} \mathrm{FDR}(\Lambda_r), \tag{11}$$

which can be used as the reference quantity for the decision of multiple hypothesis tests. For example, we can set the test level to be 0.01, i.e., choosing z_r such that $q_r^s(z) \leq 0.01$ for all $z \geq z_r$. Clearly, this rule possesses the sure screening property when n is sufficiently large. Finally, we note that other FDR methods, e.g. [9], which account for the dependence between testing p-values can also be used here for determining an appropriate threshold for marginal inclusion probabilities.

References

1. Jiang W (2007) Bayesian Variable Selection for High Dimensional Generalized Linear Models: Convergence Rates of the Fitted Densities. Ann Statist 35: 1487-1511.

2. Liang F, Song Q, Yu K (2013) Bayesian Subset Modeling for High Dimensional Generalized Linear Models. J Amer Statist Assoc, in press. doi:10.1080/01621459.2012.761942.

3. Johnson VE, Rossell D (2012) Bayesian Model Selection in High-Dimensional Settings. J Amer Statist Assoc 107: 649-660.

4. Chen MH, Schmeiser BW (1996) General hit-and-run Monte Carlo sampling for evaluating multidimensional integrals. Oper Res Lett 19: 161-169.

5. Holzmann H, Munk A, Gneiting T (2006) Identifiability of Finite Mixtures of Elliptical Distributions. Scand J Statist 33: 753-763.

6. Liang F, Zhang J (2008) Estimating FDR under general dependence using stochastic approximation. Biometrika 95: 961-977.

7. Zhang J, Liang F (2008) Convergence of stochastic approximation under irregular conditions. Statist Neerl 62: 393-403.

8. Storey JD (2002) A Direct Approach to False Discovery Rates. J Roy Statist Soc B 64: 479-498.

9. Benjamini Y, Krieger AM, Yekutieli D (2006) Adaptive Linear Step-up Procedures That Control the False Discovery Rate. Biometrika 93: 491-507.
